# Optimal dose of selective serotonin reuptake inhibitors, venlafaxine, and mirtazapine in major depression: a systematic review and dose-response meta-analysis

**DOI:** 10.1016/S2215-0366(19)30217-2

**Published:** 2019-07

**Authors:** Toshi A Furukawa, Andrea Cipriani, Philip J Cowen, Stefan Leucht, Matthias Egger, Georgia Salanti

**Affiliations:** a Department of Health Promotion and Human Behavior, and Department of Clinical Epidemiology, Graduate School of Medicine/School of Public Health, Kyoto University, Kyoto, Japan; b Department of Psychiatry, University of Oxford, Warneford Hospital, Oxford, UK; c Oxford Health NHS Foundation Trust, Warneford Hospital, Oxford, UK; d Technical University of Munich, School of Medicine, Department of Psychiatry and Psychotherapy, Munich, Germany; e Institute of Social and Preventive Medicine, University of Bern, Bern, Switzerland

## Abstract

**Background:**

Depression is the single largest contributor to non-fatal health loss worldwide. Second-generation antidepressants are the first-line option for pharmacological management of depression. Optimising their use is crucial in reducing the burden of depression; however, debate about their dose dependency and their optimal target dose is ongoing. We have aimed to summarise the currently available best evidence to inform this clinical question.

**Methods:**

We did a systematic review and dose-response meta-analysis of double-blind, randomised controlled trials that examined fixed doses of five selective serotonin reuptake inhibitors (SSRIs; citalopram, escitalopram, fluoxetine, paroxetine, and sertraline), venlafaxine, or mirtazapine in the acute treatment of adults (aged 18 years or older) with major depression, identified from the Cochrane Central Register of Controlled Trials, CINAHL, Embase, LILACS, MEDLINE, PsycINFO, AMED, PSYNDEX, websites of drug licensing agencies and pharmaceutical companies, and trial registries. We imposed no language restrictions, and the search was updated until Jan 8, 2016. Doses of SSRIs were converted to fluoxetine equivalents. Trials of antidepressants for patients with depression and a serious concomitant physical illness were excluded. The main outcomes were efficacy (treatment response defined as 50% or greater reduction in depression severity), tolerability (dropouts due to adverse effects), and acceptability (dropouts for any reasons), all after a median of 8 weeks of treatment (range 4–12 weeks). We used a random-effects, dose-response meta-analysis model with flexible splines for SSRIs, venlafaxine, and mirtazapine.

**Findings:**

28 554 records were identified through our search (24 524 published and 4030 unpublished records). 561 published and 121 unpublished full-text records were assessed for eligibility, and 77 studies were included (19 364 participants; mean age 42·5 years, SD 11·0; 7156 [60·9%] of 11 749 reported were women). For SSRIs (99 treatment groups), the dose-efficacy curve showed a gradual increase up to doses between 20 mg and 40 mg fluoxetine equivalents, and a flat to decreasing trend through the higher licensed doses up to 80 mg fluoxetine equivalents. Dropouts due to adverse effects increased steeply through the examined range. The relationship between the dose and dropouts for any reason indicated optimal acceptability for the SSRIs in the lower licensed range between 20 mg and 40 mg fluoxetine equivalents. Venlafaxine (16 treatment groups) had an initially increasing dose-efficacy relationship up to around 75–150 mg, followed by a more modest increase, whereas for mirtazapine (11 treatment groups) efficacy increased up to a dose of about 30 mg and then decreased. Both venlafaxine and mirtazapine showed optimal acceptability in the lower range of their licensed dose. These results were robust to several sensitivity analyses.

**Interpretation:**

For the most commonly used second-generation antidepressants, the lower range of the licensed dose achieves the optimal balance between efficacy, tolerability, and acceptability in the acute treatment of major depression.

**Funding:**

Japan Society for the Promotion of Science, Swiss National Science Foundation, and National Institute for Health Research.

## Introduction

Depression is the leading cause of disability worldwide.^1^ The number of people living with depression increased by around 18% between 2005 and 2015, and depression affects 322 million people, or about 4% of the world's population.^1^ Pharmacotherapy and psychotherapy are the two mainstays of depression treatment. In particular, second-generation antidepressants, including selective serotonin reuptake inhibitors (SSRIs), are the first-line options in the pharmacological management of major depression.^2^

However, there is still uncertainty about the dose dependency and optimal target dose of second-generation agents. Current practice guidelines provide conflicting recommendations: the National Institute of Health and Care Excellence guideline in UK states that no dose dependency has been established within the therapeutic range of SSRIs,^3^ whereas the American Psychiatric Association (APA) guideline recommends titration up to the maximum tolerated dose: “Initial doses should be incrementally raised as tolerated until a therapeutic dose is reached…doses of antidepressant medications should be maximized, side effects permitting.”^4^ Systematic and comprehensive reviews of the literature examining dose dependency of antidepressants should clarify the issue and inform the guideline recommendations. Unfortunately, the available reviews are few and their conclusions disagree.^5^, ^6^, ^7^ Moreover, they addressed mainly dose-efficacy relationships and gave little attention to the balance between efficacy, tolerability, and overall acceptability of treatment.

**Research in context**

**Evidence before this study**

Second-generation antidepressants, including selective serotonin reuptake inhibitors (SSRIs), are the mainstay in the pharmacological management of major depression; however, current practice guidelines provide conflicting recommendations as to their optimum target dose. The National Institute of Health and Care Excellence guideline in the UK states that no dose dependency has been established within the therapeutic range of SSRIs, whereas the American Psychiatric Association guideline recommends titration up to the maximum tolerated dose. We searched for reviews that analysed dose-response relationships for second-generation antidepressants in PubMed using the search terms “depressive disorder”, “antidepressive agents, second-generation”, and “dose-response relationship, drug”, and in the references of the identified studies, up to March 21, 2019. We identified three systematic reviews: one concluded 21–40 mg fluoxetine equivalents provided maximum efficacy, another found 40–50 mg fluoxetine-equivalent dose category offered the greatest efficacy, and the third confirmed a linearly increasing dose-efficacy relationship from placebo, to low doses, to high doses of SSRIs. Only two studies examined dose dependency for adverse effects, and only one examined dose dependency for acceptability of treatment.

**Added value of this study**

The current study is based on the largest and most comprehensive dataset of double-blind, randomised controlled trials, published and unpublished, that examined fixed doses of SSRIs, venlafaxine, or mirtazapine in the acute treatment of adults with major depression. Efficacy was dose dependent up to 20–40 mg fluoxetine equivalents for SSRIs, up to 75–150 mg for venlafaxine, and up to approximately 30 mg for mirtazapine. Above these limits, no further increase in efficacy for SSRIs or mirtazapine occurred, but there was a slight increase in efficacy for venlafaxine. There was clear dose dependency in dropouts due to adverse effects for all drugs. Consequently, the overall acceptability of treatments was optimal towards the lower end of the licensed range for SSRIs, venlafaxine, and mirtazapine. In this state-of-the art dose-response meta-analysis, dose was treated as a continuous variable, allowing greater resolution of change points and avoiding misleading categorisation of doses.

**Implications of all the available evidence**

For the majority of patients receiving SSRIs, venlafaxine, or mirtazapine, the lower range of their licensed dose will probably achieve the optimal balance between efficacy, tolerability, and acceptability. This information should inform treatment guidelines and clinical decision making in routine clinical practice.

We therefore did a dose-response meta-analysis of fixed-dose studies of commonly prescribed antidepressants for the treatment of adults with major depression,^2^ examining not only their efficacy, but also their tolerability and acceptability, to provide summative evidence to inform future guideline recommendations.

## Methods

### Search strategy and selection criteria

We included double-blind, randomised controlled trials (RCTs) comparing antidepressants among themselves or with placebo as oral monotherapy for the acute-phase treatment of adults (aged 18 years or older) of both sexes, with a primary diagnosis of major depressive disorder according to standard operationalised diagnostic criteria. Trials of antidepressants for patients with depression and a serious concomitant physical illness were excluded.^9^ This study focused on the most frequently prescribed new-generation antidepressants in the UK according to Open Prescribing,^10^ namely five SSRIs (citalopram, escitalopram, fluoxetine, paroxetine, and sertraline), venlafaxine, and mirtazapine.

The dataset was based on our 2016 network meta-analysis,^9^ which was based on searches of the Cochrane Central Register of Controlled Trials, CINAHL, Embase, LILACS, MEDLINE, MEDLINE In-Process, PsycINFO, AMED, the UK National Research Register, and PSYNDEX. We scrutinised reference lists of all relevant papers. We searched files of the national drug licensing agencies in six countries (USA, UK, Netherlands, Sweden, Japan, and Australia), the European Medicines Agency, and several trial registries for published, unpublished, and ongoing RCTs. We contacted all pharmaceutical companies marketing second-generation antidepressants and asked for supplemental unpublished information about their pre-marketing and post-marketing trials. We contacted the National Institute for Health and Care Excellence (UK), the Institut für Qualität und Wirtschaftlichkeit im Gesundheitswesen (Germany), and other relevant organisations and individuals for additional information not already identified. We used broad search terms for depression (depress* or dysthymi* or adjustment disorder* or mood disorder* or affective disorder or affective symptoms), and generic and commercial names of all antidepressants under review. We imposed no language restriction and the search was updated until Jan 8, 2016.

The complete dataset from the above search is available at Mendeley. There was no indication of small study effects, including publication bias, in this dataset.^2^ The reporting of the study followed the PRISMA guidelines.^8^ The protocol is available on the institutional websites of Kyoto University and University of Oxford.

To examine dose-dependency relationships, we included all trials that compared two or more fixed-dose treatment groups including placebo (ie, active drug with placebo, or two or more doses of an active drug with or without placebo) within a trial. We included treatment groups within and outside the licensed dose range according to international drug approval agencies.

We evaluated the risk of bias in generation of allocation sequence, allocation concealment, masking of study personnel and participants, masking of outcome assessor, attrition, and selective outcome reporting. Studies were classified as having low risk of bias if none of these domains was rated as high risk of bias and three or fewer were rated as unclear risk; moderate if one was rated as high risk of bias or none was rated as high risk of bias, but four or more were rated as unclear risk; and all other cases were assumed to have high risk of bias.^9^

At least two independent reviewers selected the studies, extracted data, and assessed risk of bias.

### Outcomes

We included the following outcomes after 8 weeks of treatment (range 4–12 weeks): treatment response (50% or greater reduction on an observer-rated scale for depression), dropouts due to adverse effects (as an index of treatment tolerability), and all-cause dropouts (interpreted as an overall index of treatment acceptability). For treatment response, we prioritised the Hamilton Depression Rating Scale,^11^ then Montgomery-Åsberg Depression Rating Scale,^12^ and if neither was used, any other validated observer-rating scale.^9^ When this outcome was not reported, we calculated response using a validated imputation method.^13^ We set the number of patients who were randomly assigned as the denominator for all outcomes, assuming that patients lost to follow-up had dropped out without experiencing response or dropout due to adverse effects.

### Comparability of dose across drugs

Dose equivalence can be defined and calculated via several methods.^14^ One method assumes the optimum doses found in double-blind, flexible-dose trials to be equivalent.^15^ In the main analyses, we used the most recent and comprehensive review of dose equivalence of antidepressants based on this method.^16^ Previous studies on dose dependency of antidepressants used similar conversion algorithms.^5^, ^7^ Where no empirical data for dose conversion were available, we assumed the daily defined dose (ie, the average maintenance dose per day calculated from the dose recommendations in each drug's product information according to WHO^17^) to be equivalent. Another method assumes the average prescribed doses in the real world for the indication to be roughly equivalent. For this purpose, we used the nationally representative Medical Expenditure Panel Survey in USA.^18^, ^19^ The dose conversion algorithms are provided in table 1.

**Table 1:** Antidepressant dose equivalence (mg) according to previous studies

	**Bollini et al (1999)**^5^	**Jakubovski et al (2016)**^7^	**Hayasaka et al (2015)**^16^	**Medical Expenditure Panel Survey (2009, 2018)**^18^, ^19^	**Defined daily dose (2006)**^17^	**Current study**
Citalopram	30	33·3	..	25·2	20	20
Escitalopram	..	16·7	9	12·3	10	9
Fluoxetine	20	20	20	31·0	20	20
Paroxetine	20	20	17	30·4	20	17
Sertraline	83	120	49·3	72·0	50	49·3
Venlafaxine	100	..	74·7	133·1	100	74·7
Mirtazapine	..	..	25·5	19·1	30	25·5

### Data analysis

We first estimated the dose dependency for the three primary outcomes by synthesising studies of all SSRIs. In this analysis we converted doses to fluoxetine equivalents using Hayasaka and colleagues'^16^ method, supplemented by the daily defined dose method. We fitted a single-stage, random-effects meta-analysis of dose-outcome model^20^ using the dosresmeta package in R.^21^ The approach estimates the association between the dose and the logarithm of risk ratio (RR) for each outcome within and across studies in a single model. We used flexible restricted cubic splines with knots at 10 mg, 20 mg, and 50 mg to have comparable numbers of studies in each quartile between placebo, knots, and the maximum dose.

We also did separate analyses for individual SSRIs, and for venlafaxine and mirtazapine.

We did the following sensitivity analyses to examine the robustness of the main findings: setting a different number of knots and at different doses; using the conversion algorithm in the previous study by Jakubovski and colleagues,^7^ or the conversion algorithm based on average doses actually prescribed for major depression;^18^, ^19^ limiting the included studies to those at low risk of bias; and taking remission as the outcome. Remission was as per the original studies, which typically defined it as scoring seven or less on the Hamilton Depression Rating Scale or ten or less on the Montgomery-Åsberg Depression Rating Scale.

The data and the analysis R code that generated the results and figures can be found online.

### Role of the funding source

The funders had no role in the design and conduct of the study; collection, management, analysis, and interpretation of the data; preparation, review, or approval of the manuscript; and decision to submit the manuscript for publication.

## Results

**Figure 1:**
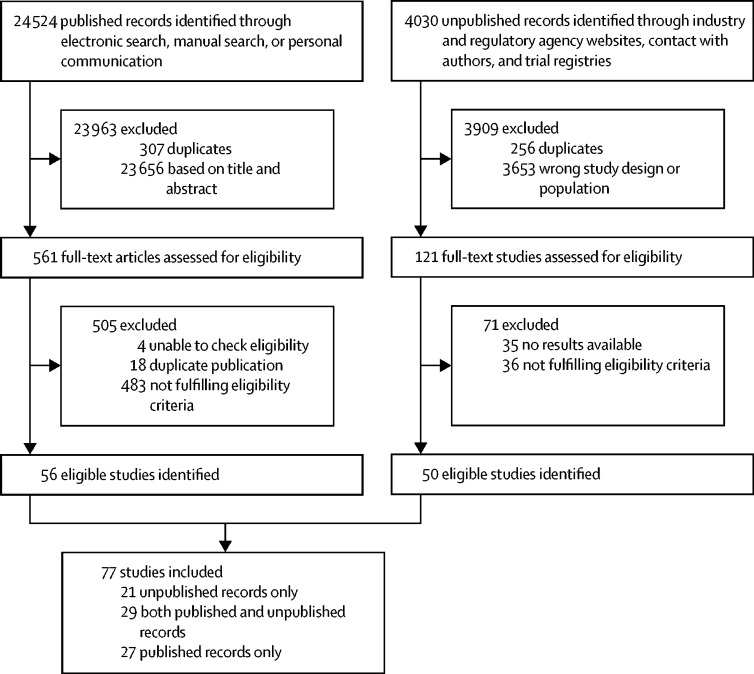
Study selection

We identified 24 524 published records through electronic search, manual search, or personal communication, and 4030 unpublished records through industry and regulatory agency websites, contact with authors, and trial registries. 561 published and 121 unpublished full-text records were assessed for eligibility, and we included 77 studies examining various fixed doses of the included drugs: 27 studies based on published articles only, 21 studies based on unpublished records only, and 29 based on both published and unpublished records (figure 1). The 77 studies included 201 treatment groups: placebo (75 treatment groups), citalopram (17 treatment groups), escitalopram (16 treatment groups), fluoxetine (27 treatment groups), mirtazapine (11 treatment groups), paroxetine (28 treatment groups), sertraline (11 treatment groups), and venlafaxine (16 treatment groups). The study year ranged between 1986 and 2013. The full references and the characteristics of the included studies are presented in the appendix.

The 77 studies included 19 364 participants (6881 were allocated to placebo and 12 483 to active drug). Their mean age was 42·5 years (SD 11·0), and 7156 (60·9%) of 11 749 participants for whom gender was reported were women. The median length of trial was 8 weeks (IQR 6–8). Nine studies had five treatment groups, 29 had four treatment groups, 30 had three treatment groups, and nine had two treatment groups. 40 (52%) were done in North America, 13 (17%) in Europe, and ten (13%) were cross-continental. 18 (23%) took place in secondary or tertiary care, four (5%) in primary care, six (78%) in both primary and secondary care, and 48 (62%) studies did not specify their study settings. The results of the risk of bias assessment are provided in the appendix. Many domains were rated as unclear and the overall risk of bias was rated as low in 21 (27%) studies, moderate in 55 (71%), and high in one (1%).

**Figure 2:**
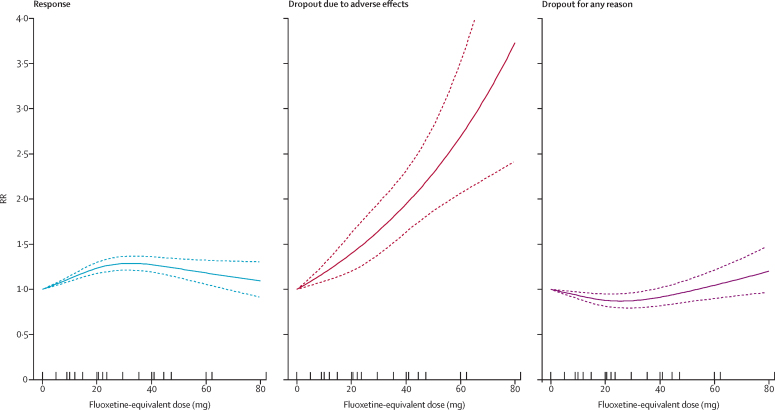
Dose-outcome relationships for selective serotonin reuptake inhibitors (99 treatment groups) RR=risk ratio. Each tick on the x-axis represents the dose examined in a treatment group. The dotted lines represent 95% CIs.

The dose-outcome relationship for treatment response, dropout due to adverse effects, and dropout for any reason for all SSRIs after the dose equivalence conversion are presented in figure 2. The RR for efficacy gradually increased from 1·0 for placebo, to 1·24 (95% CI 1·18–1·30) for 20 mg, 1·27 (1·19–1·36) for 40 mg, and then showed a flat to decreasing trend through the higher doses (ie, 41–80 mg). Above 50 mg only a few doses were examined, resulting in less precise estimates with wider CIs in the upper dose range. The association between the dose and the dropouts due to adverse effects was linear to exponential, increasing from 1·0 for placebo, to 1·94 (95% CI 1·63–2·31) at 40 mg, and to 3·73 (2·42–5·76) at the upper limit of the licensed range (80 mg). The association between the dose and the dropouts for any reason, reflecting dropouts for lack of efficacy and low tolerability, indicates optimal acceptability in the lower range between 20 mg and 40 mg. The point estimates and their 95% CIs of RRs and risk differences (RDs) for response, dropouts due to adverse effects, and dropouts for any reason at 10–80 mg of fluoxetine equivalents of SSRIs are presented in table 2.

**Table 2:** Relative risks (RRs) and risk differences (RDs) for efficacy, tolerability and acceptability at various doses of selective serotonin reuptake inhibitors (SSRIs), venlafaxine, and mirtazapine

	**Response**		**Dropouts due to adverse effects**		**Dropouts for any reason**	
	RR	RD	RR	RD	RR	RD
**SSRIs**						
10 mg	1·12 (1·09 to 1·15)	0·04 (0·03 to 0·05)	1·18 (1·09 to 1·28)	0·01 (0·00 to 0·01)	0·93 (0·89 to 0·97)	−0·02 (−0·03 to −0·01)
20 mg	1·24 (1·18 to 1·30)	0·08 (0·06 to 0·10)	1·40 (1·20 to 1·62)	0·02 (0·01 to 0·03)	0·88 (0·81 to 0·95)	−0·03 (−0·05 to −0·01)
30 mg	1·29 (1·21 to 1·36)	0·10 (0·07 to 0·13)	1·65 (1·39 to 1·96)	0·03 (0·02 to 0·04)	0·87 (0·79 to 0·96)	−0·03 (−0·05 to −0·01)
40 mg	1·27 (1·19 to 1·36)	0·09 (0·06 to 0·12)	1·94 (1·63 to 2·31)	0·04 (0·02 to 0·05)	0·91 (0·82 to 1·02)	−0·02 (−0·05 to 0·01)
60 mg	1·18 (1·06 to 1·32)	0·06 (0·01 to 0·10)	2·69 (2·06 to 3·52)	0·07 (0·04 to 0·10)	1·04 (0·90 to 1·21)	0·01 (−0·03 to 0·05)
80 mg	1·09 (0·91 to 1·30)	0·02 (−0·04 to 0·09)	3·73 (2·42 to 5·76)	0·10 (0·05 to 0·19)	1·20 (0·97 to 1·49)	0·05 (−0·01 to 0·12)
**Venlafaxine**						
37·5 mg	1·15 (1·06 to 1·25)	0·05 (0·02 to 0·09)	1·53 (1·25 to 1·87)	0·02 (0·01 to 0·03)	0·91 (0·71 to 1·17)	−0·02 (−0·07 to 0·04)
75 mg	1·31 (1·12 to 1·52)	0·11 (0·04 to 0·18)	2·24 (1·53 to 3·29)	0·05 (0·02 to 0·09)	0·85 (0·54 to 1·33)	−0·04 (−0·12 to 0·08)
150 mg	1·47 (1·24 to 1·75)	0·16 (0·08 to 0·26)	3·08 (1·95 to 4·85)	0·08 (0·04 to 0·15)	0·92 (0·64 to 1·32)	−0·02 (−0·09 to 0·08)
225 mg	1·53 (1·31 to 1·78)	0·19 (0·11 to 0·27)	3·21 (2·09 to 4·92)	0·09 (0·04 to 0·16)	1·14 (0·87 to 1·48)	0·04 (−0·03 to 0·12)
300 mg	1·58 (1·30 to 1·95)	0·20 (0·11 to 0·33)	3·31 (2·02 to 5·44)	0·09 (0·04 to 0·18)	1·42 (0·75 to 2·69)	0·11 (−0·06 to 0·42)
375 mg	1·64 (1·25 to 2·14)	0·22 (0·09 to 0·40)	3·42 (1·81 to 6·45)	0·10 (0·03 to 0·22)	1·77 (0·60 to 5·19)	0·19 (−0·10 to 1·05)
**Mirtazapine**						
7·5 mg	1·08 (1·01 to 1·16)	0·03 (0·00 to 0·06)	1·23 (1·05 to 1·46)	0·01 (0·00 to 0·02)	0·99 (0·88 to 1·11)	0·00 (−0·03 to 0·03)
15 mg	1·17 (1·02 to 1·34)	0·06 (0·01 to 0·12)	1·52 (1·09 to 2·13)	0·02 (0·00 to 0·05)	0·98 (0·78 to 1·23)	−0·01 (−0·06 to 0·06)
30 mg	1·28 (1·03 to 1·58)	0·10 (0·01 to 0·20)	2·17 (1·26 to 3·73)	0·05 (0·01 to 0·11)	0·99 (0·67 to 1·46)	0·00 (−0·08 to 0·12)
45 mg	1·16 (0·95 to 1·42)	0·06 (−0·02 to 0·15)	2·54 (1·54 to 4·21)	0·06 (0·02 to 0·13)	1·07 (0·69 to 1·64)	0·02 (−0·08 to 0·16)
60 mg	0·95 (0·65 to 1·38)	−0·02 (−0·12 to 0·13)	2·66 (1·44 to 4·92)	0·07 (0·02 to 0·16)	1·20 (0·71 to 2·05)	0·05 (−0·07 to 0·26)

RDs for efficacy, tolerability and acceptability were calculated from RRs and the average observed event rates of 35%, 4%, and 25% for response, dropouts due to side effects, and all-cause dropouts, respectively, in the included studies.

The relationships between the dose and the three outcomes for each SSRI separately are presented in the appendix. The dose-outcome curves and their 95% CIs substantially overlapped, suggesting little heterogeneity among the SSRIs in their dose-outcome relationships.

**Figure 3:**
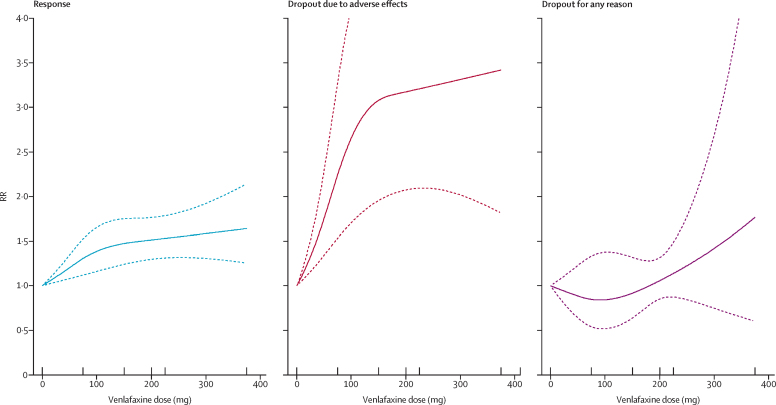
Dose-outcome relationships for venlafaxine (16 treatment groups) RR=risk ratio. Each tick on the x-axis represents the dose examined in a treatment group. The dotted lines represent 95% CIs.

**Figure 4:**
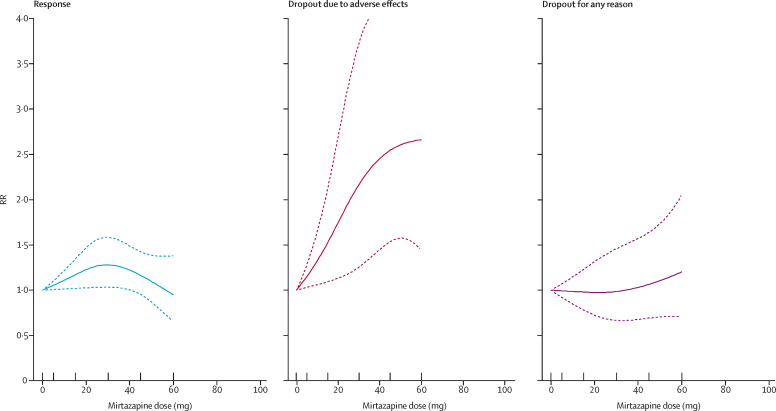
Dose-outcome relationships for mirtazapine (11 treatment groups) RR=risk ratio. Each tick on the x-axis represents the dose examined in a treatment group. The dotted lines represent 95% CIs.

The dose-outcome relationships for venlafaxine and mirtazapine are presented in Figure 3, Figure 4, respectively. The efficacy of venlafaxine increased fairly steeply up to around 75–150 mg and more modestly with higher doses (ie, 151–375 mg), whereas the efficacy of mirtazapine increased up to a dose of 30 mg and then decreased. Dropouts due to adverse effects increased steeply with increasing doses for both drugs, resulting in a dose-acceptability curve that was convex at the lower licensed range, which approximately corresponded with 20–40 mg of fluoxetine equivalents in both cases. However, studies for each individual drug were few, and the 95% CIs of the spline curves remained wide.

We examined various knots when drawing spline curves for the dose-outcome curves for SSRIs (appendix). All curves overlapped with our primary analyses. When we examined different dose-equivalence calculations among SSRIs or limited to low risk of bias studies (appendix), all the results were similar to the primary results (figure 1). The dose-remission relationship showed the same tendencies as the dose-response for SSRIs, venlafaxine, and mirtazapine (appendix).

## Discussion

We did a comprehensive dose-response meta-analysis of the most commonly prescribed antidepressants, based on the largest pool of antidepressant trials for major depression.^2^ For SSRIs, the probability of response increased up to doses between 20 mg and 40 mg of fluoxetine equivalents, with no further increase or even a slight decrease at higher doses within the licensed dose range up to 80 mg. Dropouts due to adverse effects showed a steep, linear to exponential, increase with increase in dose. Consequently, dropouts from all causes, including for lack of efficacy and for adverse effects, were lowest between 20 mg and 40 mg. Venlafaxine and mirtazapine showed slightly different dose-efficacy relationships; however, all drugs showed optimal acceptability towards the lower end of their licensed dose range.

These results are in line with psychopharmacological investigations. Using PET scans, Meyer and colleagues^22^ showed that approximately 80% serotonin transporter occupancy occurs at minimum therapeutic doses of citalopram, fluoxetine, paroxetine, sertraline, or venlafaxine, and that further increments in dose resulted in only small increases in transporter occupancy: a hyperbolic relationship typical of ligand-receptor binding.^23^ Our data also suggest that increases in transporter occupancy above 80% do not result in greater treatment efficacy. Similarly, conventional antipsychotic drugs require dopamine D_2_ receptor occupancy of about 70% to achieve a therapeutic effect; greater D_2_ occupancy does not increase efficacy, but raises the incidence of side-effects.^24^ Venlafaxine is a serotonin and noradrenaline reuptake inhibitor; however, functional noradrenaline reuptake blockade might become apparent only at higher doses (eg, 225 mg and 375 mg per day of venlafaxine).^25^ This dual action might be responsible for the observed increase in efficacy with higher doses for venlafaxine. Mirtazapine has more complex psychopharmacological properties, and the precise mechanisms underpinning its antidepressant action are less well understood. Mirtazapine is thought to increase noradrenaline and serotonin release through antagonism of central α2-adrenergic autoreceptors and heteroreceptors. It also exhibits antagonism to some serotonin (5-HT) receptor subtypes (5-HT_2A_, 5-HT_2C_, and 5-HT_3_), while overall increasing tonic activation of post-synaptic 5-HT_1A_ receptors.^26^

Earlier reviews examining the clinical dose-efficacy relationship of antidepressants have produced variable results; some of this confusion might be due to arbitrary categorisation of doses and their inconsistent naming. Based on 33 studies of new-generation and older-generation antidepressants (n=5844), Bollini and colleagues^5^ used four categories for dose (<20 mg, 21–40 mg, 41–50 mg, and >50 mg of fluoxetine equivalents) and concluded that the response rate showed a gradual increase up to 21–40 mg, with no further increase for the two high-dose categories. The inclusion of older tricyclic antidepressants limits applicability of their review to modern practices. Hieronymus and colleagues^6^ analysed individual patient data from 11 trials of citalopram, paroxetine, and sertraline (n=2859) and found that “doses below or at the lower end of the recommended dose range were superior to placebo” and “inferior to higher doses”, suggesting a linearly increasing dose-efficacy relationship for SSRIs. Within their high-dose categories corresponding with citalopram 40–60 mg, paroxetine 20–40 mg, or sertraline 100–200 mg, they found no indication of dose dependency. However, their category of low dose included subtherapeutic doses (citalopram 10 mg or paroxetine 10 mg) as well as therapeutic doses (citalopram 20 mg and sertraline 50 mg), which might explain why the low-dose category did less well than the high-dose category in their analysis.

In 2016, Jakubovski and colleagues^7^ found a statistically significantly greater response for high doses in a meta-regression analysis of 40 studies comparing SSRIs with placebo (n=10 039); an accompanying editorial^27^ concluded, “there is a modest but clear dose-response effect for SSRIs.” However, when the findings were categorised into less than 20 mg, 20–39 mg, 40–50 mg, and more than 50 mg fluoxetine equivalents, the authors found the 40–50 mg dose category offered the greatest efficacy (ie, there was no general linear relationship between dose and efficacy over the whole dose range). Moreover, a majority of their included studies were flexible-dose studies and they took the maximum of the flexible range as the dose representing the treatment groups.

Our dose-dependency curves were based on models using splines, which treated dose as a continuous variable, and shed new light on the existing evidence. Our results for dose-efficacy relationships for SSRIs are largely in line with Bollini and colleagues.^5^ Hieronymus and colleagues'^6^ observation that there was a stepwise increase in efficacy from placebo, to low dose, to higher dose of SSRIs might be considered compatible with our finding of dose response up to 20–40 mg of fluoxetine equivalents; however, their categorisation of doses did not capture the change points identified in our analyses. Jakubovski and colleagues'^7^ conclusion that 40–50 mg of fluoxetine equivalents offered the greatest efficacy might be overstated if we take into account that most of their included studies used flexible-dose regimens and the prescribed doses could be significantly lower than 40–50 mg.

Two studies examined the relationships between doses and side-effects. Bollini and colleagues^5^ found a monotonic increase in adverse event rates, from 20% to 50%, as the dose increased, from zero to more than 50 mg fluoxetine equivalents. Jakubovski and colleagues^7^ found a monotonic dose relationship for dropouts due to side-effects, but an almost flat relationship for all-cause dropouts. These findings might be confounded by the inclusion of both fixed-dose and flexible-dose studies. By contrast, by focusing on fixed-dose studies and using flexible spline curves, we found a linear to exponential relationship between dose and dropouts due to side-effects, and a curvilinear relationship between dose and all-cause dropouts for SSRIs.

Our study is not without limitations. First, the findings mainly pertain to patients with major depression who were judged eligible for placebo-controlled trials. For patients who suffer from physical comorbidities or for older (ie, age ≥65 years) patients, the optimal dose might be lower than 20–40 mg fluoxetine equivalents. In this context, the positive dose-efficacy association through zero to 30 mg of fluoxetine equivalents is clinically relevant. Second, the fixed-dose regimen might be regarded as not reflecting clinical practice, especially when no or rapid titration scheme is used, and might overestimate withdrawal due to adverse effects. Tolerability might then confound efficacy because interventions with high dropouts are likely to show lower endpoint efficacy because the majority of patients leave the study early and therefore have less time to improve; however, strict examination of dose dependency is possible only with fixed-dose studies. Third, there is some uncertainty about how best to calculate dose equivalency among antidepressants. We used the most comprehensive empirically derived conversion algorithms,^16^ which were similar to the ones used in previous systematic reviews on this topic.^5^, ^7^ We tested our results via sensitivity analyses that used alternative conversion algorithms and confirmed the primary results. Fourth, spline curves for individual drugs were based on a few studies, resulting in wide CIs. We therefore meta-analysed all SSRIs because they share a key therapeutic mechanism. The curves for individual SSRIs provided little evidence of heterogeneity. For venlafaxine and mirtazapine, the numbers of available trials were few and the resultant wide CIs, particularly regarding tolerability and acceptability, warn against overinterpreting the results. Fifth, we used dropouts for side-effects from the acute-phase treatment as an index of tolerability: these numbers do not necessarily reflect rare but severe adverse events, nor do they include long-term side-effects, including withdrawal symptoms. Lastly, the search date for the relevant studies could be considered old; however, an update search for eligible trials in PubMed on March 21, 2019 revealed only one additional eligible study with a small sample size (55 patients randomly assigned to paroxetine 10 mg and 49 to placebo),^28^ which would not change the results of our analysis with 19 364 patients.

Our study has a number of strengths. First, we used state-of-the art dose-response meta-analysis, and treated dose as a continuous variable, allowing greater resolution of change points and avoiding misleading categorisation of doses. Second, we examined dose dependency not only for efficacy but also for tolerability and acceptability. Third, our study is based on the largest and most comprehensive dataset of fixed-dose, double-blind, randomised controlled trials, published or unpublished, of second-generation antidepressants in the acute-phase treatment of major depression. The included numbers of studies and participants were two to eight times larger than the previous analyses. We analysed not only SSRIs, but also two widely prescribed non-SSRI antidepressants, venlafaxine and mirtazapine. Our findings will have clinical implications, especially for practitioners or countries where fluoxetine is routinely prescribed at doses of 40 mg or more.

In conclusion, our analyses showed dose dependency in efficacy up to around 20–40 mg fluoxetine equivalents, beyond which there is no further increase in efficacy for SSRIs or mirtazapine, but a possibly slight increase for venlafaxine; a clear dose dependency in dropouts due to adverse effects for all drugs through the examined dose range; and the overall acceptability of treatments appears to be optimal towards the lower end of the licensed range. We therefore conclude that for the majority of patients receiving an SSRI, venlafaxine, or mirtazapine for the acute-phase treatment of their major depressive episode, the lower range of the licensed dose will probably achieve the optimal balance between efficacy, tolerability, and acceptability. Clinical guidelines need to incorporate these findings.

For more on **Open Prescribing** see https://openprescribing.net/
