# Supplementary material for: Optimal dose of selective serotonin reuptake inhibitors, venlafaxine, and mirtazapine in major depression: a systematic review and dose-response meta-analysis
Source: Lancet Psychiatry. 2019 Jul;6(7):601–9. doi: 10.1016/S2215-0366(19)30217-2 (PMC6586944; doi:10.1016/S2215-0366(19)30217-2)
Supplement: Supplementary appendix [file mmc1.pdf]

THE LANCET
Psychiatry

Supplementary appendix

This appendix formed part of the original submission and has been peer reviewed. We post it as supplied by the authors.

Supplement to: Furukawa TA, Cipriani A, Cowen PJ, Leucht S, Egger M, Salanti G. Optimal dose of selective serotonin reuptake inhibitors, venlafaxine and mirtazapine in major depression: systematic review and dose-response meta-analysis. *Lancet Psychiatry* 2019; published online June 6. http://dx.doi.org/10.1016/S2215-0366(19)30217-2.

Optimal dose of selective serotonin reuptake inhibiors, venlafaxine and mirtazapine in major depression: Systematic review and dose-response meta-analysis

Toshi A. Furukawa

Andrea Cipriani

Philip J. Cowen

Stefan Leucht

Matthias Egger

Georgia Salanti

Supplementary Appendix

Contents

1 Reference list of included studies 3

2 Characteristics of included studies 11

3 Risk of bias table of included studies 19

4 Dose-outcome curves 22

1 *Reference list of included studies*

1. Alvarez 2012 (11492A, NCT00839423)

- Alvarez E, Perez V, Dragheim M, Loft H, Artigas F. A double-blind, randomized, placebo-controlled, active reference study of Lu AA21004 in patients with major depressive disorder. Int J Neuropsychopharmacol. 2012;15(5):589-600.
- https://clinicaltrials.gov/ct2/show/NCT00839423

2. Binnemann 2008 (NCT00143091)

- Binneman B, Feltner D, Kolluri S, Shi Y, Qiu R, Stiger T. A 6-week randomized, placebo-controlled trial of CP-316,311 (a selective CRH1 antagonist) in the treatment of major depression. Am J Psychiatry. 2008;165(5):617-20.

3. Bjerkenstedt 2005

- Bjerkenstedt L1, Edman GV, Alken RG, Mannel M. Hypericum extract LI 160 and fluoxetine in mild to moderate depression: a randomized, placebo-controlled multi-center study in outpatients. Eur Arch Psychiatry Clin Neurosci. 2005 Feb;255(1):40-7.

4. Brunoni 2012

- Brunoni AR, Valiengo L, Baccaro A, Zanão TA, de Oliveira JF, Goulart A, Boggio PS, Lotufo PA, Benseñor IM, Fregni F. The sertraline vs. electrical current therapy for treating depression clinical study: results from a factorial, randomized, controlled trial. JAMA Psychiatry. 2013;70(4):383-91.

5. Burke 2002 (SCT-MD-01)

- Burke WJ, Gergel I, Bose A. Fixed-dose trial of the single isomer SSRI escitalopram in depressed outpatients. J Clin Psychiatry. 2002 Apr;63(4):331-6

6. CL3-20098-022

- http://www.ema.europa.eu/docs/en_GB/document_library/EPAR_-_Public_assessment_report/human/000916/WC500038315.pdf

7. CL3-20098-023

- http://www.ema.europa.eu/docs/en_GB/document_library/EPAR_-_Public_assessment_report/human/000915/WC500046226.pdf

8. CL3-20098-024

- http://www.ema.europa.eu/docs/en_GB/document_library/EPAR_-_Public_assessment_report/human/000916/WC500038315.pdf

9. CN104-054 (FDA)

- https://digitalcollections.ohsu.edu/concern/etds/3484zh30d

10. Corrigan 2000

- Corrigan MH, Denahan AQ, Wright CE, Ragual RJ, Evans DL. Comparison of pramipexole, fluoxetine, and placebo in patients with major depression. Depress Anxiety 2000;11(2):58-65.

11. Detke 2004 (HMAY Study Group A)

- Detke MJ, Wiltse CG, Mallinckrodt CH, McNamara RK, Demitrack MA, Bitter I. Duloxetine in the acute and long-term treatment of major depressive disorder: a placebo- and paroxetine-controlled trial. Eur Neuropsychopharmacol 2004; 14: 457–70.
- http://www.lillytrials.com/results/Cymbalta.pdf

12. Dunner 1992 (PAR 29060.09)

- Dunner DL, Dunbar GC. Optimal dose regimen for paroxetine. J Clin Psychiatry. 1992;53 Suppl:21-6.
- http://www.gsk-clinicalstudyregister.com/study/29060/009#rs

13. Edwards 1989 (MD/PAR/009 PAR-276)

- Edwards JG, Goldie A. Placebo-controlled trial of paroxetine in depressive illness. Human Psychopharmacology 1993;8:203-209
- http://www.gsk-clinicalstudyregister.com/study/29060/276?search=compound&compound=paroxetine#rs

14. Faber 1995 (SER 103 FDA)

- Fabre L, Abuzzahab F, Amin M, Claghorn J, Mendels J, Dubé S, Small JG. Sertraline in major depression: double-blind comparison with placebo. Biol Psychiatry 1995;38:592-602.
- https://digitalcollections.ohsu.edu/concern/etds/nk322d61z

15. Fava 2005

- Fava M, Alpert J, Nierenberg AA, Mischoulon D, Otto MW, Zajecka J, Murck H, Rosenbaum JF. A Double-blind, randomized trial of St John's wort, fluoxetine, and placebo in major depressive disorder. J Clin Psychopharmacol. 2005 Oct;25(5):441-7.

16. Feighner 1999 (Study 91206 FDA)

- Feighner JP1, Overø K. Multicenter, placebo-controlled, fixed-dose study of citalopram in moderate-to-severe depression. J Clin Psychiatry. 1999 Dec;60(12):824-30.

17. Frank 2004

- Frank MG, Hendricks SE, Burke WJ, Johnson DR. Clinical response augments NK cell activity independent of treatment modality: a randomized double-blind placebo controlled antidepressant trial. Psychol Med. 2004 Apr;34(3):491-8.

18. Gastpar 2006

- Gastpar M, Singer A, Zeller K. Comparative efficacy and safety of a once-daily dosage of hypericum extract STW3-VI and citalopram in patients with moderate depression: a double-blind, randomised, multicentre, placebo-controlled study. Pharmacopsychiatry. 2006 Mar;39(2):66-75.

19. Goldstein 2004a (HMAT - Study Group A, ID#4091)

- http://www.lillytrials.com/results/Cymbalta.pdf

20. Goldstein 2004b (HMAT - Study Group B, ID#4091)

- Goldstein DJ, Lu Y, Detke MJ, Wiltse C, Mallinckrodt C, Demitrack MA. Duloxetine in the treatment of depression: a double-blind placebo-controlled comparison with paroxetine. J Clin Psychopharmacol. 2004; 24: 389–99.

21. Griebel 2012 (Study DF15878, NCT00358631)

- Griebel G, Beeské S, Stahl SM. The vasopressin V(1b) receptor antagonist SSR149415 in the treatment of major depressive and generalized anxiety disorders: results from 4 randomized, double-blind, placebo-controlled studies. J Clin Psychiatry. 2012 Nov;73(11):1403-11.
- https://clinicaltrials.gov/ct2/show/NCT00358631

22. Griebel 2012b (Study DF15879, NCT00361491)

- Griebel G, Beeské S, Stahl SM. The vasopressin V(1b) receptor antagonist SSR149415 in the treatment of major depressive and generalized anxiety disorders: results from 4 randomized, double-blind, placebo-controlled studies. J Clin Psychiatry. 2012 Nov;73(11):1403-11.
- https://clinicaltrials.gov/ct2/show/NCT00361491

23. Heiligenstein 1994

- Heiligenstein J, Faries D, Rush A, Andersen J, Pande A, Roffwarg H, Dunner D, Gillin J, James S, Lahmeyer H. Latency to rapid eye movement sleep as a predictor of treatment response to fluoxetine and placebo in nonpsychotic depressed outpatients. Psychiatry Res. 1994;52:327-39.

24. Higuchi 2014 (NCT01441440)

- Higuchi T, Kamijima K, Nakagome K, Itamura R, Asami Y, Kuribayashi K, Imaeda T. A randomized, double-blinded, placebo-controlled study to evaluate the efficacy and safety of venlafaxine extended release and a long-term extension study for patients with major depressive disorder in Japan. Int Clin Psychopharmacol. 2016 Jan;31(1):8-19.
- https://clinicaltrials.gov/ct2/show/NCT01441440

25. Hirayasu 2011a

- Hirayasu Y. A dose-response study of escitalopram in patients with major depressive disorder: a placebo-controlled, double-blind study. Jpn J Clin Psychopharmacol 2011;14:871-882.

26. Hirayasu 2011b

- Hirayasu Y. A dose-response and non-inferiority study evaluating the efficacy and safety of escitalopram in patients with major depressive disorder. Jpn J Clin Psychopharmacol 2011;14:883-899.

27. Hunter 2010 (Study 1)

- Hunter AM, Muthén BO, Cook IA, Leuchter AF. Antidepressant response trajectories and quantitative electroencephalography (QEEG) biomarkers in major depressive disorder. J Psychiatr Res. 2010 Jan;44(2):90-8.

28. Hunter 2010 (Study 2)

- Hunter AM, Muthén BO, Cook IA, Leuchter AF. Antidepressant response trajectories and quantitative electroencephalography (QEEG) biomarkers in major depressive disorder. J Psychiatr Res. 2010 Jan;44(2):90-8.

29. Hunter 2010 (Study 3)

- Hunter AM, Muthén BO, Cook IA, Leuchter AF. Antidepressant response trajectories and quantitative electroencephalography (QEEG) biomarkers in major depressive disorder. J Psychiatr Res. 2010 Jan;44(2):90-8.

30. Jefferson 2000 (29060/785)

- Jefferson J, Griest J. A double-blind, placebo controlled, fixed-dosage study comparing the efficacy and tolerability of paroxetine CR and citalopram to placebo in the treatment of Major Depressive Disorder with anxiety. American College of Neuropsychopharmacology, 39th Annual Meeting 12/11/2000 San Juan, Puerto Rico.
- http://www.gsk-clinicalstudyregister.com/study/29060/785#csr

31. Kasper 2005a (Study 99024)

- Kasper S, de Swart H, Friis Andersen H. Escitalopram in the treatment of depressed elderly patients. Am J Geriatr Psychiatry. 2005 Oct;13(10):884-91.

32. Kasper 2012 (NCT00807248)

- Kasper S, Ebert B, Larsen K, Tonnoir B. Combining escitalopram with gaboxadol provides no additional benefit in the treatment of patients with severe major depressive disorder. Int J Neuropsychopharmacol. 2012 Jul;15(6):715-25.
- https://clinicaltrials.gov/ct2/show/NCT00807248

33. Keller 2006a (Study059, NCT00035009)

- Keller M, Montgomery S, Ball W, Morrison M, Snavely D, Liu G, Hargreaves R, Hietala J, Lines C, Beebe K, Reines S. Lack of efficacy of the substance p (neurokinin1 receptor) antagonist aprepitant in the treatment of major depressive disorder. Biol Psychiatry. 2006 Feb 1;59(3):216-23.
- https://clinicaltrials.gov/ct2/show/NCT00035009

34. Keller 2006b (Study061, NCT00035295)

- Keller M, Montgomery S, Ball W, Morrison M, Snavely D, Liu G, Hargreaves R, Hietala J, Lines C, Beebe K, Reines S. Lack of efficacy of the substance p (neurokinin1 receptor) antagonist aprepitant in the treatment of major depressive disorder. Biol Psychiatry. 2006 Feb 1;59(3):216-23.
- https://clinicaltrials.gov/ct2/show/NCT00035295?term=NCT00035295&rank=1

35. Keller 2006c (Study062, NCT00048607)

- Keller M, Montgomery S, Ball W, Morrison M, Snavely D, Liu G, Hargreaves R, Hietala J, Lines C, Beebe K, Reines S. Lack of efficacy of the substance p (neurokinin1 receptor) antagonist aprepitant in the treatment of major depressive disorder. Biol Psychiatry. 2006 Feb 1;59(3):216-23.

- https://clinicaltrials.gov/ct2/show/NCT00048607?term=NCT00048607&rank=1

36. Khan 1998

- Khan A, Upton GV, Rudolph RL, Entsuah R, Leventer SM. The use of venlafaxine in the treatment of major depression and major depression associated with anxiety: a dose-response study. Venlafaxine Investigator Study Group. J Clin Psychopharmacol. 1998 Feb;18(1):19-25.

37. Kinoshita 2009

- Kinoshita T. A double-blind, placebo controlled study of a new antidepressant, mirtazapine, in depressed patients. Jpn J Clin Psychopharmacol 2009;12:289-306.

38. Kramer 1998

- Kramer MS, Cutler N, Feighner J, Shrivastava R, Carman J, Sramek JJ, Reines SA, Liu G, Snavely D, Wyatt-Knowles E, Hale JJ, Mills SG, MacCoss M, Swain CJ, Harrison T, Hill RG, Hefti F, Scolnick EM, Cascieri MA, Chicchi GG, Sadowski S, Williams AR, Hewson L, Smith D, Carlson EJ, Hargreaves RJ, Rupniak NM. Distinct mechanism for antidepressant activity by blockade of central substance P receptors. Science. 1998 Sep 11;281(5383):1640-5.

39. Lam 1995

- Lam RW, Gorman CP, Michalon M, Steiner M, Levitt AJ, Corral MR, Watson GD, Morehouse RL, Tam W, Joffe RT. Multicenter, placebo-controlled study of fluoxetine in seasonal affective disorder. Am J Psychiatry. 1995;152(12):1765-70.

40. Learned 2012b Study2 (NCT00420641)

- Learned S, Graff O, Roychowdhury S, Moate R, Krishnan KR, Archer G, Modell JG, Alexander R, Zamuner S, Lavergne A, Evoniuk G, Ratti E. Efficacy, safety, and tolerability of a triple reuptake inhibitor GSK372475 in the treatment of patients with major depressive disorder: two randomized, placebo- and active-controlled clinical trials. J Psychopharmacol. 2012 May;26(5):653-62.
- https://clinicaltrials.gov/ct2/show/NCT00420641

41. Loo 2002 (CL2-014)

- Lôo H, Hale A, D'haenen H. Determination of the dose of agomelatine, a melatoninergic agonist and selective 5-HT(2C) antagonist, in the treatment of major depressive disorder: a placebo-controlled dose range study. Int Clin Psychopharmacol. 2002 Sep;17(5):239-47.

42. Lopez-Rodriguez 2004

- Rodriguez JL, Lopez Butron MA, Vargas Terrez BE, Villamil Salcedo V. Estudio doble ciego con antidepressivo, psicoterapia breve y placebo en pacientes con depression leve a moderada. Salud Mental 2004;27:53-61.

43. Mathews 2015 (NCT01473381)

- Mathews M, Gommoll C, Chen D, Nunez R, Khan A. Efficacy and safety of vilazodone 20 and 40 mg in major depressive disorder: a randomized, double-blind, placebo-controlled trial. Int Clin Psychopharmacol. 2015;30(2):67-74.

- https://clinicaltrials.gov/ct2/show/NCT01473381

44. Miller 1989 (MDUK/29060/III/82/006, PAR-274, PAR UK 06 - FDA)

- Miller SM, Naylor GJ, Murtagh M, Winslow G. A double-blind comparison of paroxetine and placebo in the treatment of depressed patients in a psychiatric outpatient clinic. Acta Psychiatr Scand Suppl. 1989;350:143-4
- https://digitalcollections.ohsu.edu/concern/etds/9s161642r

45. Montgomery 1992 (Study 89303 FDA)

- http://www.accessdata.fda.gov/drugsatfda_docs/nda/98/020822a.cfm

46. Moreno 2005

- Moreno RA, Teng CT, Almeida KM, Tavares Junior H. Hypericum perforatum versus fluoxetine in the treatment of mild to moderate depression: a randomized double-blind trial in a Brazilian sample. Rev Bras Psiquiatr. 2006 Mar;28(1):29-32.

47. MY-1008/BRL-029060/2/CPMS-076

- https://www.gsk-clinicalstudyregister.com/study/29060/076#rs

48. NCT00822744 (EudraCT Number2008-001718-26)

- https://clinicaltrials.gov/ct2/show/NCT00822744
- https://www.clinicaltrialsregister.eu/ctr-search/trial/2008-001718-26/SK

49. NCT01020799

- https://clinicaltrials.gov/ct2/show/NCT01020799

50. NCT01808612

- https://clinicaltrials.gov/ct2/show/NCT01808612

51. Nierenberg 2007 (F1J-MC-HMCR, NCT00073411, Pigott 2007)

- Nierenberg AA, Greist JH, Mallinckrodt CH, et al. Duloxetine versus escitalopram and placebo in the treatment of patients with major depressive disorder: onset of antidepressant action, a non-inferiority study. Curr Med Res Opin 2007; 23: 401–16.
- http://www.lillytrials.com/results/Cymbalta.pdf
- https://clinicaltrials.gov/ct2/show/NCT00073411

52. NKD20006 (NCT00048204)

- http://www.gsk-clinicalstudyregister.com/study/NKD20006#ps
- https://clinicaltrials.gov/ct2/show/NCT00048204

53. PAR 279 MDUK

- http://www.gsk-clinicalstudyregister.com/study/29060/012_3?search=compound&compound=paroxetine#rs

54. Perahia 2006 (HMAY - Study Group B)

- Perahia DG, Wang F, Mallinckrodt CH, Walker DJ, Detke MJ. Duloxetine in the treatment of major depressive disorder: a placebo- and paroxetine-controlled trial. Eur Psychiatry 2006; 21: 367–78.

- http://www.lillytrials.com/results/Cymbalta.pdf

55. Perahia 2008b (HMCQ, NCT00067912)

- Perahia DGS, Pritchett Y, Kajdasz D, Bauer M, Jain R, Russell JM, Walker DJ, Spencer KA, Froud DM, Raskin J, Thase ME. A randomized, double-blind comparison of duloxetine and venlafaxine in the treatment of patients with major depressive disorder. Journal of Psychiatric Research. 2008;42(1):22-34.
- https://clinicaltrials.gov/ct2/show/NCT00067912

56. Rapaport 2009 (BRL-29060/874, NCT00067444)

- Rapaport MH, Lydiard RB, Pitts CD, Schaefer D, Bartolic EI, Iyengar M, Carfagno M, Lipschitz A. Low doses of controlled-release paroxetine in the treatment of late-life depression: a randomized, placebo-controlled trial. J Clin Psychiatry. 2009 Jan;70(1):46-57.
- https://www.clinicaltrials.gov/ct2/show/NCT00067444

57. Rudolph 1998 (VEN 600A-203 (FDA)

- Rudolph RL, Fabre LF, Feighner JP, Rickels K, Entsuah R, Derivan AT. A randomized, placebo-controlled, dose-response trial of venlafaxine hydrochloride in the treatment of major depression. J Clin Psychiatry. 1998 Mar;59(3):116-22.
- https://digitalcollections.ohsu.edu/concern/etds/pk02c992m

58. SCT-MD-35 (NCT00109044)

- https://clinicaltrials.gov/ct2/show/NCT00668525

59. SCT-MD-49 (NCT00668525)

- https://clinicaltrials.gov/ct2/show/NCT00668525

60. SER 101 (FDA)

- https://digitalcollections.ohsu.edu/concern/etds/nk322d61z

61. SER 310 (FDA)

- https://digitalcollections.ohsu.edu/concern/etds/nk322d61z

62. Sramek 1995

- Sramek JJ, Kashkin K, Jasinsky O, Kardatzke D, Kennedy S, Cutler NR. Placebo-controlled study of ABT-200 versus fluoxetine in the treatment of major depressive disorder. Depression 1995;3:199-203.

63. Study 62a (FDA) - MILD depression (Dunlop 1990)

- Dunlop SR, Dornseif BE, Wernicke JR, Potvin JH. Pattern analysis shows beneficial effect of fluoxetine treatment in mild depression. Psychopharmacology bulletin. 1990;26(2):173-80.
- https://digitalcollections.ohsu.edu/concern/etds/5h73pw36j

64. Study 62b (FDA) - MODERATE depression (Wernicke1987)

- Wernicke JF, Dunlop SR, Dornseif BE, Zerbe RL. Fixed-dose fluoxetine therapy for depression. Psychopharmacology Bulletin. 1987;23(1):164-68.
- https://digitalcollections.ohsu.edu/concern/etds/5h73pw36j

65. Study 89306 (FDA)

- http://www.accessdata.fda.gov/drugsatfda_docs/nda/98/020822a_medr_P2.pdf

66. Study F1J-MC-HMAQ - Study Group B (starting page in the pdf document: 147)

- http://www.lillytrials.com/results/Cymbalta.pdf

67. Trivedi 2004 (29060/810)

- Trivedi MH, Pigotti TA, Perera P, Dillingham KE, Carfagno ML, Pitts CD. Effectiveness of low doses of paroxetine controlled release in the treatment of major depressive disorder. J Clin Psychiatry. 2004;65(10):1356-64.
- http://www.gsk-clinicalstudyregister.com/study/29060/810?search=study&#rs

68. VEN 600A-313 (FDA)

- https://digitalcollections.ohsu.edu/concern/etds/pk02c992m

69. VEN XR 367 (FDA)

- https://digitalcollections.ohsu.edu/concern/etds/z029p503q

70. Wade 2002 (ESC Study 99001 - FDA)

- Wade A, Michael Lemming O, Bang Hedegaard K. Escitalopram 10 mg/day is effective and well tolerated in a placebo-controlled study in depression in primary care. Int Clin Psychopharmacol. 2002;17(3):95-102.
- http://www.lundbeck.com/upload/trials/files/pdf/completed/99001_CTRS_final_30Dec2005.pdf

71. Wernicke 1988

- Wernicke JF, Dunlop SR, Dornseif BE, Bosomworth JC, Humbert M. Low-dose fluoxetine therapy for depression. Psychopharmacology Bulletin. 1988;24(1):183-88.

72. Yevtushenko 2007

- Yevtushenko VY, Belous AI, Yevtushenko YG, Gusinin SE, Buzik OJ, Agibalova TV. Efficacy and tolerability of escitalopram versus citalopram in major depressive disorder: a 6-week, multicenter, prospective, randomized, double-blind, active-controlled study in adult outpatients. Clin Ther. 2007 Nov;29(11):2319-32.

73. 003-008 (FDA)

- https://digitalcollections.ohsu.edu/concern/etds/p8418n49j

74. 003-042

- https://digitalcollections.ohsu.edu/concern/etds/p8418n49j

75. 003-048

- https://digitalcollections.ohsu.edu/concern/etds/p8418n49j

76. 245 (EMD 68 843-010)

- http://www.accessdata.fda.gov/drugsatfda_docs/nda/2011/022567Orig1s000MedR.pdf

77. 246 (SB 659746-003)

- http://www.accessdata.fda.gov/drugsatfda_docs/nda/2011/022567Orig1s000MedR.pdf

2 *Characteristics of included studies*

Study Name	Year	No. of arms included in MA	No of total study arms	Drug	Sponsored	No. randomised	Mean age (SD)	% Female	Dosing Schedule	Mean Dose Delivered	Baseline Severity Scale	Mean Baseline Severity	Total length of trial (weeks)	Timing of outcome assessment (weeks)	Diagnostic Criteria	Recruitment	Multi/ Single Centre	Treatment Setting	Patient Status	Placebo Run in	Rescue Medication
Alvarez 2012 (11492A, NCT00839423)	2012	4	4	placebo	No	105	42 (10.9)	0.66	Fixed	0	HAMD 24	29.70	6	6	DSM-IV	Cross-Continental	Multi-center	Secondary/Tertiary care	Outpatients only	Unclear	Yes
				venlafaxine	No	114	45 (10.3)	0.54	Fixed	225	HAMD24	29.40									
Binnemann 2008 (NCT00143091)	2008	2	3	sertraline	Yes	43	49	*	Fixed	100	HAMD 17	23.30	6	6	DSM-IV	Cross-Continental	Multi-center	Unclear	Outpatients only	Yes	Unclear
				placebo	No	39	49	*	Fixed	0	HAMD 17	23.55									
Bjerkenstedt 2005	2005	2	3	fluoxetine	No	57	50.4 (11.6)	*	Fixed	20	HAMD 21	23.80	6	6	DSM-IV	Europe	Multi-center	Primary care	Outpatients only	Yes	Unclear
				placebo	No	58	51.4 (11.8)	*	Fixed	0	HAMD 21	25.20									
Brunoni 2012	2012	2	4	sertraline	No	30	41 (12)	*	Fixed	50	MADRS	30.5	6	6	DSM-IV	South America	Single center	Secondary/ Tertiary care	Outpatients only	No	Yes
				placebo	No	30	46.4 (14)	*	Fixed	0	MADRS	30.76									
Burke 2002 (SCT-MD-01)	2002	4	4	escitalopram	Yes	124	40.6 (12.3)	*	Fixed	10	HAMD unspecified	24.30	8	8	DSM-IV	North America	Multi-center	Unclear	Outpatients only	Yes	Unclear
				escitalopram	Yes	128	39.6 (12.1)	*	Fixed	20	HAMD unspecified	25.80									
				citalopram	Yes	127	40 (11.5)	*	Fixed	40	HAMD unspecified	25.90									
				placebo	No	127	40.3 (10.6)	*	Fixed	0	HAMD unspecified	25.80									
CL3-20098-022	2001	2	3	fluoxetine	no	137	42.9 (11.3)	0.67	Fixed	20	HAMD 17	27.50	24	6	DSM-IV	Europe	Multi-center	Secondary/Tertiary care	Both	Yes	No
				placebo	No	149	43 (9.5)	0.67	Fixed	0	HAMD 17	28.00									
CL3-20098-023	2001	2	3	paroxetine	No	138	40.9 (11)	0.75	Fixed	20	HAMD 17	26.10	24	6	DSM-IV	Cross-Continental	Multi-center	Secondary/Tertiary care	Both	No	No
				placebo	No	137	41.2 (10.1)	0.75	Fixed	0	HAMD 17	26.00									
CL3-20098-024	2002	2	4	fluoxetine	No	148	41.3 (10)	0.73	Fixed	20	HAMD 17	26.50	24	6	DSM-IV	Cross-Continental	Multi-center	Secondary/Tertiary care	Both	No	Unclear
				placebo	No	158	41.6 (11.2)	0.73	Fixed	0	HAMD 17	26.90									
CN104-054 (FDA)	unpublished	2	3	fluoxetine	No	*	*	0.68	Fixed	20	*	*	8	8	Unclear	North America	Multi-center	Unclear	Outpatients	Unclear	Unclear
				placebo	No	*	*	0.68	Fixed	0	*	*									
Corrigan 2000	2000	2	5	fluoxetine	No	35	42	*	Fixed	20	HAMD 17	22.00	8	8	DSM-III-R	North America	Multi-center	Secondary/Tertiary care	Unclear	Yes	Unclear
				placebo	No	35	42	*	Fixed	0	HAMD 17	20.80									
	2004	2	4	paroxetine	No	86	42 (10.6)	*	Fixed	20	HAMD 17	20.26	8	8	DSM-IV	Unspecified	Multi-center	Unclear	Outpatients only	Yes	Unclear

Detke 2004 (HMAY Study Group A)				placebo	No	93	43.67 (12.2)	*	Fixed	0	HAMD 17	19.86									
Dunner 1992 (PAR 29060.09)	1992	5	5	paroxetine	Yes	105	40.6	*	Fixed	10	HAMD 21	25.21	6	6	DSM-III	North America	Multi-center	Other/Unclear	Outpatients only	Unclear	Unclear
				paroxetine	Yes	106	41.6	*	Fixed	20	HAMD 21	25.19									
				paroxetine	Yes	104	41.6	*	Fixed	30	HAMD 21	25.18									
				paroxetine	Yes	106	41.4	*	Fixed	40	HAMD 21	25.03									
				placebo	No	53	40.9	*	Fixed	0	HAMD 21	24.50									
Edwards 1989 (MD/PAR/009 PAR-276)	1989	2	2	paroxetine	Yes	21	45.1	0.55	Fixed	30	HAMD 17	26.80	6	6	DSM-III	Europe	Single center	Secondary/Tertiary care	Outpatients only	Unclear	Yes
				placebo	No	21	43.3	0.57	Fixed	0	HAMD 17	25.50									
Faber 1995 (SER 103 FDA)	1995	4	4	sertraline	Yes	95	37	0.51	Fixed	50	HAMD17	24.80	6	6	DSM-III	North America	Multi-center	Other/Unclear	Outpatients only	Yes	Yes
				sertraline	Yes	92	37	0.57	Fixed	100	HAMD 17	24.90									
				sertraline	Yes	91	38	0.63	Fixed	200	HAMD 17	25.70									
				placebo	No	91	37	0.44	Fixed	0	HAMD 17	25.30									
Fava 2005	2005	2	3	placebo	No	43	37.8 (12)	*	Fixed	0	HAMD 17	19.90	12	8	DSM-IV	North America	Multi-center	Secondary/Tertiary care	Outpatients only	Yes	Unclear
				fluoxetine	No	47	36.7 (9.6)	*	Fixed	20	HAMD 17	19.60									
Feighner 1999 (Study 91206 FDA)	1999	4	5	citalopram	Yes	131	38	0.63	Fixed	10	HAMD21	25.1	6	6	DSM-III-R	North America	Multi-center	Unclear	Outpatients only	Yes	Yes
				citalopram	Yes	130	39	0.66	Fixed	20	HAMD 21	24.2									
				citalopram	Yes	131	39	0.60	Fixed	40	HAMD 21	24.4									
				citalopram	Yes	129	38	0.53	Fixed	60	HAMD 21	24.5									
				placebo	No	129	38	0.55	Fixed	0	HAMD 21	24.6									
Frank 2004	2004	2	2	citalopram	Yes	8	39.12 (6.7)	0.25	Fixed	20	*	*	4	4	DSM-IV	North America	Single center	Secondary/Tertiary care	Outpatients only	No	Unclear
				placebo	No	8	41.5 (7.1)	0.75	Fixed	0	*	*									
Gastpar 2006	2006	2	3	citalopram	No	127	49.3 (10.7)	0.65	Fixed	20	HAMD unspecified	21.80	6	6	DSM-IV	Europe	Multi-center	Primary care	Outpatients only	Unclear	Unclear
				placebo	No	130	49.4 (12.7)	0.73	Fixed	0	HAMD unspecified	22.00									
Goldstein 2004a (HMAT - Study Group A, ID#4091)	2004	2	4	placebo	No	90	43.18 (14.5)	*	Fixed	0	HAMD 17	17.79	8	8	DSM-IV	North America	Multi-center	Unclear	Outpatients only	Yes	Unclear
				paroxetine	Yes	89	44.43 (14.7)	*	Fixed	20	HAMD 17	17.97									
Goldstein 2004b (HMAT - Study Group B, ID#4091)	2004	2	4	placebo	No	89	40.14 (12.9)	*	Fixed	0	HAMD 17	17.19	8	8	DSM-IV	North America	Multi-center	Unclear	Outpatients only	Yes	Unclear
				paroxetine	No	87	40.25 (11.0)	*	Fixed	20	HAMD 17	17.65									

Griebel 2012 (Study DF15878) (NCT00358631)	2012	2	4	escitalopram	No	84	40.8 (10.7)	0.62	Fixed	10	HAMD 17	23.40	8	8	DSM-IV	North America	Multi-center	Unclear	Outpatients only	Yes	Unclear
				placebo	No	76	41.2 (12.4)	0.63	Fixed	0	HAMD 17	23.00									
Griebel 2012b (Study DF15879) (NCT00361491)	2012	2	4	paroxetine	No	80	40 (13.2)	0.70	Fixed	20	HAMD 17	24.90	8	8	DSM-IV	Cross-Continental	Multi-center	Unclear	Outpatients only	Yes	Unclear
				placebo	No	79	40.1 (10.6)	0.73	Fixed	0	HAMD 17	24.80									
Heiligenstein 1994	1994	2	2	fluoxetine	Yes	46	41.6 (10.9)	*	Fixed	20	HAMD 17	21.10	8	8	DSM-III-R	North America	Multi-center	Secondary/Tertiary care	Outpatients only	Yes	Yes
				placebo	No	43	39.2 (9.8)	*	Fixed	0	HAMD 17	21.60									
Higuchi 2014 (NCT01441440)	2016	3	3	venlafaxine	Yes	174	38.4 (11.9)	0.52	Fixed	75	HAMD 17	*	8	8	DSM-IV	Asia	Multi-center	Unclear	Outpatients only	Unclear	Unclear
				placebo	No	184	38.6 (11.1)	0.51	Fixed	0	HAMD 17	*									
Hirayasu 2011a	2011	3	3	escitalopram	Yes	105	35.9 (11.2)	*	Fixed	20	HAMD 17	22.20	8	8	DSM-IV	Asia	Multi-center	Both	Outpatients only	No	Yes
				escitalopram	Yes	100	33.1 (9)	*	Fixed	10	HAMD 17	22.30									
				placebo	No	105	34.7 (8.6)	*	Fixed	0	HAMD 17	22.50									
Hirayasu 2011b	2011	4	4	placebo	No	124	36.4 (10.8)	*	Fixed	0	MADRS	29.00	8	8	DSM-IV	Asia	Multi-center	Both	Outpatients only	Yes	Yes
				escitalopram	Yes	121	36.3 (9.7)	*	Fixed	10	MADRS	29.40									
				escitalopram	Yes	119	35.7 (9.8)	*	Fixed	20	MADRS	29.80									
Hunter 2010 (Study 1)	2010	2	2	fluoxetine	Yes	14	42.4 (12.6)	0.68	Fixed	20	HAMD 17	22.10	8	8	DSM-IV	North America	Multi-center	Unclear	Outpatients only	Yes	No
				placebo	No	14	42.4 (12.6)	0.68	Fixed	0	HAMD 17	22.10									
Hunter 2010 (Study 2)	2010	2	2	venlafaxine	Yes	17	44.7 (14)	0.64	Fixed	150	HAMD 17	22.30	8	8	DSM-IV	North America	Multi-center	Unclear	Outpatients only	Yes	No
				placebo	No	16	44.7 (14)	0.64	Fixed	0	HAMD 17	22.30									
Hunter 2010 (Study 3)	2010	2	2	venlafaxine	Yes	18	38.1 (12.5)	0.55	Fixed	150	HAMD 17	21.50	8	8	DSM-IV	North America	Multi-center	Unclear	Outpatients only	Yes	No
				placebo	No	15	38.1 (12.5)	0.55	Fixed	0	HAMD 17	21.50									
Jefferson 2000 (29060/785)	2000	5	5	placebo	No	105	40.2 (11.5)	*	Fixed	0	MADRS	31.7	6	6	DSM-IV	North America	Multi-center	Unclear	Outpatients only	No	Unclear
				paroxetine	Yes	96	41.2 (12.3)	*	Fixed	12.5	MADRS	31.7									
				paroxetine	Yes	103	41.5 (12.1)	*	Fixed	25	MADRS	31.4									
				citalopram	Yes	107	38.4 (11.3)	*	Fixed	20	MADRS	31.4									
				citalopram	Yes	100	39.6 (11.1)	*	Fixed	40	MADRS	31.0									
Kasper 2005a (Study 99024)	2005	3	3	escitalopram	Yes	174	75 (7)	*	Fixed	10	MADRS	28.20	8	8	DSM-IV	Europe	Multi-center	Both	Both	Yes	Unclear
				fluoxetine	No	164	75 (7)	*	Fixed	20	MADRS	28.50									

				placebo	No	180	75 (7)	*	Fixed	0	MADRS	28.60									
Kasper 2012 (NCT00807248)	2012	2	4	escitalopram	Yes	140	41.6 (12.6)	0.69	Fixed	20	MADRS	35.40	8	8	DSM-IV	Europe	Multi-center	Secondary/Tertiary care	Both	Unclear	No
				placebo	No	71	42.6 (11.6)	0.75	Fixed	0	MADRS	34.70									
Keller 2006a (Study 059, NCT00035009)	2006	2	3	paroxetine	No	154	39	0.68	Fixed	20	HAMD 17	27.50	8	8	DSM-IV	North America	Multi-center	Unclear	Outpatients only	Unclear	Unclear
				placebo	No	155	41.4	0.61	Fixed	0	HAMD 17	27.70									
Keller 2006b (Study 061) (NCT00035295)	2006	2	4	paroxetine	No	143	42.2	0.75	Fixed	20	HAMD 17	27.60	8	8	DSM-IV	North America	Multi-center	Unclear	Outpatients only	Unclear	Unclear
				placebo	No	145	41.1	0.74	Fixed	0	HAMD 17	27.30									
Keller 2006c (Study 062) (NCT00048607)	2006	2	4	paroxetine	No	164	40.6	0.64	Fixed	20	HAMD 17	28.30	8	8	DSM-IV	Cross-Continental	Multi-center	Unclear	Outpatients only	Unclear	Unclear
				placebo	No	161	41.3	0.71	Fixed	0	HAMD 17	28.30									
Khan 1998	1998	4	4	venlafaxine	Yes	85	43.3	0.68	Fixed	75	HAMD 21	24.30	12	8	DSM-III-R	North America	Multi-center	Unclear	Outpatients only	yes	unclear
				venlafaxine	Yes	90	40	0.64	Fixed	150	HAMD 21	24.50									
				venlafaxine	Yes	83	43.6	0.60	Fixed	200	HAMD 21	24.80									
				placebo	No	95	40.2	0.61	Fixed	0	HAMD 21	25.10									
Kinoshita 2009	2009	4	4	mirtazapine	Yes	70	39.4 (10.9)	*	Fixed	15	HAMD 17	23.20	6	6	DSM-IV	Asia	Multi-center	Both	Both	No	No
				mirtazapine	Yes	70	37.7 (10.8)	*	Fixed	30	HAMD 17	22.50									
				mirtazapine	Yes	71	41.6 (12.5)	*	Fixed	45	HAMD 17	22.10									
				placebo	No	70	39.9 (12.8)	*	Fixed	0	HAMD 17	22.50									
Kramer 1998	1998	2	3	paroxetine	Unclear	72	*	*	Fixed	20	HAMD 21	*	6	6	DSM-IV	North America	Multi-center	Other/Unclear	Outpatients only	Unclear	Unclear
				placebo	No	70	*	*	Fixed	0	HAMD 24	*									
Lam 1995	1995	2	2	fluoxetine	Yes	36	40.9 (8.6)	*	Fixed	20	HAMD 21	18.70	5	5	DSM-III-R	North America	Multi-center	Secondary/Tertiary care	Outpatients only	Yes	No
				placebo	No	32	38.1 (9.7)	*	Fixed	0	HAMD 21	18.30									
Learned 2012b Study2 (NCT00420641)	2012	2	3	paroxetine	Yes	166	44.4 (10.9)	0.33	Fixed	30	MADRS	30.30	10	8	DSM-IV	Cross-Continental	Multi-center	Other/Unclear	Outpatients only	Unclear	Unclear
				placebo	No	156	41.8 (10.9)	0.25	Fixed	0	MADRS	31.80									
Loo 2002 (CL2-014)	2002	2	5	placebo	No	139	42.3	0.67	Fixed	0	HAMD 17	27.4	8	8	DSM-IV	Europe	Multi-center	Both	Other/Unclear	Yes	Yes
				paroxetine	No	147	42.3	0.67	Fixed	20	HAMD 17	27.3									
LopezRodriguez 2004	2004	2	4	fluoxetine	Unclear	10	31.9	*	Fixed	20	HAMD 21	17.27	20	9	DSM-IV	South America	Single center	Primary care	Outpatients only	No	Unclear
				placebo	No	10	31.9	*	Fixed	0	HAMD 21	17.27									
Mathews 2015 (NCT01473381)	2015	2	4	placebo	No	290	42 (13)	0.56	Fixed	0	MADRS	31.30	10	8	DSM-IV	North America	Multi-center	Other/Unclear	Outpatients only	Unclear	Yes

				citalopram	No	289	42.6 (12.6)	0.59	Fixed	40	MADRS	31.10									
Miller 1989 (MDUK/29060/III/82/006 (PAR-274) PAR UK 06 - FDA)	1989	2	2	paroxetine	Yes	22	41.6 (13.2)	*	Fixed	30	HAMD 21	23.70	4	4	Feighner	Europe	Single center	Other/Unclear	Outpatients only	Unclear	Unclear
				placebo	No	25	43.3 (11.8)	*	Fixed	0	HAMD 21	24.20									
Montgomery 1992 (Study 89303 FDA)	unpublished	3	3	citalopram	Yes	70	*	*	Fixed	20	HAMD 17	24.29	6	6	DSM-III-R	*	Multi-center	Other/Unclear	Both	Yes	Yes
				citalopram	Yes	64	*	*	Fixed	40	HAMD 17	22.98									
				placebo	No	66	*	*	Fixed	0	HAMD 17	23.72									
Moreno 2005	2005	2	3	fluoxetine	No	20	37.7 (9.9)	0.83	Fixed	20	HAMD 21	15.41	8	8	DSM-IV	South America	Single center	Secondary/Tertiary care	Outpatients only	Yes	Yes
				placebo	No	26	45.9 (10.8)	0.83	Fixed	0	HAMD 21	16.49									
MY-1008/BRL-029060/2/CPMS-076	unpublished	2	3	paroxetine	Yes	4	49.3	1.00	Fixed	30	HAMD 21	*	4	4	DSM-IV	Europe	Multi-center	Other/Unclear	Other/Unclear	Unclear	Unclear
				placebo	No	4	46.5	0.50	Fixed	0	HAMD 21	*									
NCT00822744 (EudraCT Number2008-001718-26)	unpublished	2	5	escitalopram	Unclear	103	*	*	Fixed	10	HAMD 17	*	8	8	DSM-IV	Cross-Continental	Multi-center	Other/Unclear	Other/Unclear	Unclear	Unclear
				placebo	No	106	*	*	Fixed	0	HAMD 17	*									
NCT01020799	unpublished	2	3	escitalopram	No	50	36.22 (11.3)	0.44	Fixed	20	HAMD unspecified	24.40	4	4	DSM-IV	North America	Multi-center	Other/Unclear	Other/Unclear	Unclear	Unclear
				placebo	No	99	41.77 (10.0)	0.49	Fixed	0	HAMD unspecified	24.60									
NCT01808612	unpublished	3	3	fluoxetine	Yes	169	40.5 (11.2)	0.49	Fixed	20	HAMD 21	*	6	6	*	Asia	Multi-center	Other/Unclear	Outpatients only	Unclear	Unclear
				fluoxetine	Yes	84	39.58 (10.6)	0.40	Fixed	40	HAMD 21	*									
				placebo	No	260	38.54 (11.6)	0.50	Fixed	0	HAMD 21	*									
Nierenberg 2007 (F1J-MC-HMCR, NCT00073411, Pigott 2007)	2007	2	3	escitalopram	No	274	43.3 (13)	*	Fixed	10	HAMD 17	17.80	8	8	DSM-IV	North America	Multi-center	Secondary/Tertiary care	Outpatients only	Yes	Unclear
				placebo	No	137	42.5 (12.3)	*	Fixed	0	HAMD 17	17.70									
NKD20006 (NCT00048204)	unpublished	2	3	paroxetine	Yes	125	37.9 (11.9)	0.63	Fixed	20	HAMD 17	24.70	8	8	DSM-IV	North America	Multi-center	Other/Unclear	Outpatients only	Yes	No
				placebo	No	125	38 (11.3)	0.56	Fixed	0	HAMD 17	24.50									
PAR 279 MDUK	unpublished	2	3	paroxetine	Yes	19	39.7 (18.2)	0.68	Fixed	30	HAMD unspecified	22.80	6	6	*	Europe	Multi-center	Other/Unclear	Other/Unclear	Yes	Unclear
				placebo	No	10	51.6 (17.3)	0.70	Fixed	0	HAMD unspecified	22.30									
Perahia 2006 (HMAY - Study Group B)	2006	2	4	paroxetine	No	97	45.81 (10.6)	*	Fixed	20	HAMD 17	21.03	8	8	DSM-IV	Unspecified	Multi-center	Other/Unclear	Outpatients only	Yes	Unclear
				placebo	No	99	44.67 (10.1)	*	Fixed	0	HAMD 17	20.58									
	2008	2	3	venlafaxine	No	169	42.46 (12.4)	0.68	Fixed	75	HAMD 17	22.51	6	6	DSM-IV	Cross-Continental	Multi-center	Other/Unclear	Outpatients only	Unclear	Unclear

Perahia 2008b (HMCQ, NCT00067912)				venlafaxine	No	171	40.64 (11.4)	0.62	Fixed	150	HAMD 17	22.24									
Rapaport 2009 (BRL-29060/874) (NCT00067444)	2009	3	3	paroxetine	Yes	168	67 (6.10)	*	Fixed	12.5	HAMD 17	22.25	10	10	DSM-IV	North America	Multi-center	Other/Unclear	Outpatients only	Yes	Unclear
				paroxetine	Yes	177	67 (6.56)	*	Fixed	25	HAMD 17	22.74									
				placebo	No	180	68 (6.7)	*	Fixed	0	HAMD 17	22.41									
Rudolph 1998 (VEN 600A-203 (FDA)	1998	4	4	venlafaxine	Yes	89	43.6 (10.9)	0.40	Fixed	75	HAMD 21	26.00	6	6	DSM-III	North America	Multi-center	Secondary/Tertiary care	Outpatients only	Yes	Yes
				venlafaxine	Yes	89	43.1 (9.61)	0.43	Fixed	225	HAMD 21	26.00									
				venlafaxine	Yes	88	43.8 (10.3)	0.33	Fixed	375	HAMD 21	24.90									
				placebo	No	92	41.5 (11.3)	0.32	Fixed	0	HAMD 21	25.30									
SCT-MD-35 (NCT00109044)	unpublished	2	4	escitalopram	YES	138	39.6 (11.7)	*	Fixed	4	MADRS	29.80	8	8	DSM-IV	North America	Multi-center	Other/Unclear	Outpatients only	Unclear	Unclear
				placebo	No	135	43.3 (13.5)	*	Fixed	0	MADRS	30.3									
SCT-MD-49 (NCT00668525)	unpublished	3	3	escitalopram	Yes	325	41.4 (12.3)	0.64	Fixed	10	HAMD 24	29.30	8	8	DSM-IV	North America	Multi-center	Other/Unclear	Other/Unclear	Yes	Unclear
				escitalopram	Yes	332	40.4 (11.9)	0.69	Fixed	28	HAMD 24	29.30									
				placebo	No	220	42.3 (12.7)	0.63	Fixed	*	HAMD 24	28.90									
SER 101 (FDA)	unpublished	4	5	sertraline	Yes	26	*	*	Fixed	50	HAMD 17	*	4	4	DSM-III	North America	Multi-center	Secondary/Tertiary care	Inpatients only	Yes	Unclear
				sertraline	Yes	24	*	*	Fixed	100	HAMD 17	*									
				sertraline	Yes	23	*	*	Fixed	200	HAMD 17	*									
				placebo	No	26	*	*	Fixed	0	HAMD 17	*									
SER 310 (FDA)	unpublished	4	4	sertraline	Yes	33	*	*	Fixed	50	*	*	4	4	DSM-III	Europe	Multi-center	Secondary/Tertiary care	Inpatients only	Yes	Yes
				sertraline	Yes	35	*	*	Fixed	100	*	*									
				sertraline	Yes	37	*	*	Fixed	200	*	*									
				placebo	No	34	*	*	Fixed	0	*	*									
Sramek 1995	1995	2	3	fluoxetine	No	72	33.9	0.60	Fixed	20	HAMD 24	28.20	9	9	DSM-III-R	North America	Single center	Other/Unclear	Other/Unclear	Yes	Unclear
				placebo	No	72	33.9	0.60	Fixed	0	HAMD 24	27.50									
Study 62a (FDA) - MILD depression (Dunlop 1990)	1990	4	4	fluoxetine	Yes	104	38.31	0.59	Fixed	60	HAMD 21	17.20	6	6	DSM-III	*	Multi-center	Other/Unclear	Outpatients only	Yes	Unclear
				fluoxetine	Yes	105	39.8	0.56	Fixed	40	HAMD 21	16.83									
				fluoxetine	Yes	107	39.97	0.62	Fixed	20	HAMD 21	16.99									
				placebo	No	56	38.96	0.50	Fixed	0	HAMD 21	17.41									
Study 62b (FDA) - MODERATE	1987	4	4	fluoxetine	Yes	105	39.93	0.58	Fixed	60	HAMD 21	24.20	6	6	DSM-III	*	Multi-center	Other/Unclear	Outpatients only	Yes	Unclear

depression (Wernicke 1987)				fluoxetine	Yes	103	41.15	0.56	Fixed	40	HAMD 21	24.19									
				fluoxetine	Yes	100	39.4	0.60	Fixed	20	HAMD 21	24.72									
				placebo	No	48	38.56	0.50	Fixed	0	HAMD 21	24.25									
Study 89306 (FDA)	unpublished	3	3	citalopram	Yes	88	*	*	Fixed	20	MADRS	32.70	6	6	DSM-III-R	*	Multi-center	Both	Both	Yes	Yes
				citalopram	Yes	97	*	*	Fixed	40	MADRS	31.30									
				placebo	No	89	*	*	Fixed	0	MADRS	33.14									
Study F1J-MC-HMAQ - Study Group B	unpublished	3	3	placebo	No	75	41.39 (11.8)	*	Fixed	0	HAMD 17	18.32	10	8	DSM-IV	North America	Multi-center	Other/Unclear	Outpatients only	Yes	Unclear
				fluoxetine	No	37	39.65 (12.7)	*	Fixed	20	HAMD 17	20.03									
Trivedi 2004 (29060/810)	2004	2	3	placebo	No	149	38.4 (12.1)	*	Fixed	0	HAMD 17	23.8	8	8	DSM-IV	North America	Multi-center	Other/Unclear	Other/Unclear	Yes	Unclear
				paroxetine	Yes	156	38.6 (10.8)	*	Fixed	12.5	HAMD 17	23.2									
				paroxetine	Yes	154	39.4 (10.8)	*	Fixed	25	HAMD 17	23.5									
VEN 600A-313 (FDA)	unpublished	3	4	venlafaxine	Yes	76	38	0.70	Fixed	75	HAMD 21	25.57	6	6	DSM-III-R	North America	Unclear	Other/Unclear	Outpatients only	Yes	Yes
				venlafaxine	Yes	82	39	0.61	Fixed	200	HAMD 21	25.57									
				placebo	No	79	38	0.69	Fixed	0	HAMD 21	25.39									
VEN XR 367 (FDA)	unpublished	4	4	venlafaxine	Yes	83	*	0.70	Fixed	75	HAMD 21	26.49	8	8	DSM-III-R	Europe	Multi-center	Other/Unclear	Outpatients only	Yes	Yes
				venlafaxine	Yes	82	*	0.61	Fixed	150	HAMD 21	27.11									
				paroxetine	No	81	*	0.54	Fixed	20	HAMD 21	26.13									
				placebo	No	83	*	0.67	Fixed	0	HAMD 21	26.60									
Wade 2002 (ESC Study 99001 - FDA)	2002	2	2	placebo	No	189	40 (12)	0.78	Fixed	0	MADRS	28.70	8	8	DSM-IV	Cross-Continental	Multi-center	Primary care	Outpatients only	Yes	Unclear
				escitalopram	Yes	191	41 (11)	0.74	Fixed	10	MADRS	29.20									
Wernicke 1988	1988	4	4	placebo	No	77	39	0.61	Fixed	0	HAMD unspecified	25.9	6	6	DSM-III	North Amereica	Multi-center	Other/Unclear	Other/Unclear	Yes	Unclear
				fluoxetine	Yes	94	39	0.61	Fixed	5	HAMD unspecified	25.5									
				fluoxetine	Yes	91	39	0.61	Fixed	20	HAMD unspecified	25.0									
				fluoxetine	Yes	92	39	0.61	Fixed	40	HAMD unspecified	25.4									
Yevtushenko 2007	2007	2	3	citalopram	No	111	34.8 (6.4)	0.58	Fixed	10	MADRS	35.40	6	6	DSM-IV	Europe	Multi-center	Secondary/Tertiary care	Outpatients only	Unclear	Yes
				citalopram	No	110	35.1 (6.5)	0.56	Fixed	20	MADRS	35.70									
003-008 (FDA)	unpublished	5	5	mirtazapine	Yes	30	41	0.70	Fixed	5	HAMD21	25.3	6	6	DSM-III	North America	Single center	Other/Unclear	Outpatients only	Yes	Unclear
				mirtazapine	Yes	30	38	0.63	Fixed	15	HAMD 21	26.00									
				mirtazapine	Yes	30	40	0.71	Fixed	30	HAMD 21	25.50									

				mirtazapine	Yes	30	43	0.62	Fixed	60	HAMD21	25.0									
				placebo	No	30	39	0.61	Fixed	0	HAMD 21	25.80									
003-042	unpublished	4	4	mirtazapine	Yes	70	41 (10.8)	0.71	Fixed	15	HAMD 21	25.30	8	8	DSM-IV	North America	Multi-center	Other/Unclear	Outpatients only	Yes	Unclear
				mirtazapine	Yes	70	39.8 (10.2)	0.56	Fixed	30	HAMD 21	24.70									
				mirtazapine	Yes	70	42.2 (12.9)	0.60	Fixed	45	HAMD 21	25.00									
				placebo	No	70	40.8 (10.8)	0.66	Fixed	0	HAMD 21	25.90									
003-048	unpublished	3	3	mirtazapine	Yes	210	41.4 (12.1)	0.60	Fixed	45	HAMD 17	22.30	8	8	DSM-IV	North America	Multi-center	Other/Unclear	Other/Unclear	Yes	Unclear
				fluoxetine	No	210	40.8 (11.6)	0.58	Fixed	60	HAMD 17	22.80									
				placebo	No	72	40.2 (11.5)	0.61	Fixed	0	HAMD 17	23.00									
245 (EMD 68 843-010)	unpublished	3	5	fluoxetine	No	92	*	*	Fixed	20	HAMD 17	23.50	8	8	DSM-IV	North America	Multi-center	Other/Unclear	Outpatients only	Yes	Unclear
				placebo	No	99	*	*	Fixed	0	HAMD 17	23.40									
246 (SB 659746-003)	unpublished	2	4	citalopram	No	117	*	*	Fixed	20	HAMD 17	23.10	8	8	DSM-IV	North America	Multi-center	Other/Unclear	Outpatients only	Yes	Unclear
				placebo	No	129	*	*	Fixed	0	HAMD 17	23.30									

3 *Risk of bias table of included studies*

For details about the rating criteria, see published protocol (Furukawa et al., BMJ Open 2016). 'Stated not tested' refers to the mention of double-blind procedures used in the methods section of a paper, but maintenance of blinding is not tested.

StudyID		Sequence_generation	Allocation_concealment	Blinding_participant	Blinding_therapist	Blinding_assessor	Selective_reporting_bias	Attrition_bias
1.	Alvarez 2012	Low risk of bias	Low risk of bias	Stated but not tested	Stated but not tested	Stated but not tested	Low risk of bias	High risk of bias
2.	Binnemann 2008	Unclear risk of bias	Unclear risk of bias	Stated but not tested	Stated but not tested	Unclear risk of bias	Low risk of bias	Unclear risk of bias
3.	Bjerkenstedt 2005	Unclear risk of bias	Unclear risk of bias	Stated but not tested	Stated but not tested	Unclear risk of bias	Low risk of bias	Low risk of bias
4.	Brunoni 2012	Low risk of bias	Low risk of bias	Low risk of bias	Stated but not tested	Unclear risk of bias	Unclear risk of bias	Low risk of bias
5.	Burke 2002	Unclear risk of bias	Unclear risk of bias	Stated but not tested	Stated but not tested	Unclear risk of bias	Low risk of bias	Unclear risk of bias
6.	CL3-20098-022	Low risk of bias	Low risk of bias	Stated but not tested	Stated but not tested	Unclear risk of bias	Low risk of bias	High risk of bias
7.	CL3-20098-023	Low risk of bias	Low risk of bias	Stated but not tested	Stated but not tested	Unclear risk of bias	Low risk of bias	Low risk of bias
8.	CL3-20098-024	Low risk of bias	Low risk of bias	Stated but not tested	Stated but not tested	Unclear risk of bias	Low risk of bias	Low risk of bias
9.	CN104-054	Unclear risk of bias	Unclear risk of bias	Stated but not tested	Stated but not tested	Unclear risk of bias	High risk of bias	Unclear risk of bias
10.	Corrigan 2000	Unclear risk of bias	Unclear risk of bias	Stated but not tested	Stated but not tested	Unclear risk of bias	Low risk of bias	High risk of bias
11.	Detke 2004	Unclear risk of bias	Unclear risk of bias	Stated but not tested	Stated but not tested	Unclear risk of bias	Low risk of bias	Low risk of bias
12.	Dunner 1992	Unclear risk of bias	Unclear risk of bias	Stated but not tested	Stated but not tested	Unclear risk of bias	Low risk of bias	Unclear risk of bias
13.	Edwards 1989	Unclear risk of bias	Unclear risk of bias	Low risk of bias	Stated but not tested	Unclear risk of bias	Low risk of bias	High risk of bias
14.	Faber 1995	Low risk of bias	Unclear risk of bias	Stated but not tested	Stated but not tested	Unclear risk of bias	Low risk of bias	High risk of bias
15.	Fava 2005	Unclear risk of bias	Unclear risk of bias	Stated but not tested	Stated but not tested	Unclear risk of bias	Low risk of bias	Unclear risk of bias
16.	Feighner 1999	Unclear risk of bias	Unclear risk of bias	Stated but not tested	Stated but not tested	Unclear risk of bias	Low risk of bias	High risk of bias
17.	Frank 2004	Unclear risk of bias	Unclear risk of bias	Stated but not tested	Stated but not tested	Unclear risk of bias	High risk of bias	Unclear risk of bias
18.	Gastpar 2006	Low risk of bias	Unclear risk of bias	Stated but not tested	Stated but not tested	Unclear risk of bias	Low risk of bias	Low risk of bias
19.	Goldstein 2004a	Unclear risk of bias	Unclear risk of bias	Stated but not tested	Stated but not tested	Unclear risk of bias	Low risk of bias	Unclear risk of bias
20.	Goldstein 2004b	Unclear risk of bias	Unclear risk of bias	Stated but not tested	Stated but not tested	Unclear risk of bias	Low risk of bias	Unclear risk of bias
21.	Griebel 2012	Unclear risk of bias	Unclear risk of bias	Stated but not tested	Stated but not tested	Unclear risk of bias	Low risk of bias	Unclear risk of bias
22.	Griebel 2012b	Unclear risk of bias	Unclear risk of bias	Stated but not tested	Stated but not tested	Unclear risk of bias	Low risk of bias	Unclear risk of bias
23.	Heiligenstein 1994	Low risk of bias	Unclear risk of bias	Stated but not tested	Stated but not tested	Unclear risk of bias	Low risk of bias	Unclear risk of bias

24.	Higuchi 2014	Unclear risk of bias	Unclear risk of bias	Stated but not tested	Stated but not tested	Stated but not tested	Unclear risk of bias	Low risk of bias
25.	Hirayasu 2011a	Unclear risk of bias	Unclear risk of bias	Stated but not tested	Stated but not tested	Stated but not tested	Low risk of bias	Low risk of bias
26.	Hirayasu 2011b	Unclear risk of bias	Unclear risk of bias	Stated but not tested	Stated but not tested	Stated but not tested	Low risk of bias	Low risk of bias
27.	Hunter 2010 (Study 1)	Unclear risk of bias	Unclear risk of bias	Stated but not tested	Stated but not tested	Unclear risk of bias	High risk of bias	Unclear risk of bias
28.	Hunter 2010 (Study 2)	Unclear risk of bias	Unclear risk of bias	Stated but not tested	Stated but not tested	Unclear risk of bias	High risk of bias	Unclear risk of bias
29.	Hunter 2010 (Study 3)	Unclear risk of bias	Unclear risk of bias	Stated but not tested	Stated but not tested	Unclear risk of bias	High risk of bias	Unclear risk of bias
30.	Jefferson 2000	Unclear risk of bias	Unclear risk of bias	Stated but not tested	Stated but not tested	Unclear risk of bias	Low risk of bias	High risk of bias
31.	Kasper 2005a	Unclear risk of bias	Unclear risk of bias	Stated but not tested	Stated but not tested	Unclear risk of bias	Low risk of bias	High risk of bias
32.	Kasper 2012	Low risk of bias	Unclear risk of bias	Stated but not tested	Stated but not tested	Stated but not tested	Low risk of bias	High risk of bias
33.	Keller 2006a	Unclear risk of bias	Unclear risk of bias	Stated but not tested	Stated but not tested	Unclear risk of bias	Unclear risk of bias	Unclear risk of bias
34.	Keller 2006b	Unclear risk of bias	Unclear risk of bias	Stated but not tested	Stated but not tested	Unclear risk of bias	Unclear risk of bias	Unclear risk of bias
35.	Keller 2006c	Unclear risk of bias	Unclear risk of bias	Stated but not tested	Stated but not tested	Unclear risk of bias	Low risk of bias	Unclear risk of bias
36.	Khan 1998	Unclear risk of bias	Unclear risk of bias	Stated but not tested	Stated but not tested	Unclear risk of bias	Low risk of bias	Unclear risk of bias
37.	Kinoshita 2009	Unclear risk of bias	Unclear risk of bias	Stated but not tested	Stated but not tested	Stated but not tested	Low risk of bias	Unclear risk of bias
38.	Kramer 1998	Unclear risk of bias	Unclear risk of bias	Stated but not tested	Stated but not tested	Unclear risk of bias	Low risk of bias	Unclear risk of bias
39.	Lam 1995	Unclear risk of bias	Unclear risk of bias	Stated but not tested	Stated but not tested	Unclear risk of bias	Low risk of bias	Unclear risk of bias
40.	Learned 2012b Study2	Unclear risk of bias	Unclear risk of bias	Stated but not tested	Stated but not tested	Unclear risk of bias	Low risk of bias	Unclear risk of bias
41.	Loo 2002	Unclear risk of bias	Unclear risk of bias	Stated but not tested	Stated but not tested	Unclear risk of bias	Low risk of bias	Unclear risk of bias
42.	LopezRodriguez 2004	Unclear risk of bias	Unclear risk of bias	Stated but not tested	Stated but not tested	Stated but not tested	Unclear risk of bias	Unclear risk of bias
43.	Mathews 2015	Low risk of bias	Unclear risk of bias	Stated but not tested	Stated but not tested	Unclear risk of bias	Low risk of bias	Unclear risk of bias
44.	Miller 1989	Unclear risk of bias	Unclear risk of bias	Stated but not tested	Stated but not tested	Unclear risk of bias	Unclear risk of bias	High risk of bias
45.	Montgomery 1992	Unclear risk of bias	Unclear risk of bias	Stated but not tested	Stated but not tested	Unclear risk of bias	Low risk of bias	Unclear risk of bias
46.	Moreno 2005	Unclear risk of bias	Unclear risk of bias	Stated but not tested	Stated but not tested	Unclear risk of bias	Unclear risk of bias	Unclear risk of bias
47.	MY-1008/CPMS-076	Unclear risk of bias	Unclear risk of bias	Stated but not tested	Stated but not tested	Unclear risk of bias	Unclear risk of bias	Unclear risk of bias
48.	NCT00822744	Unclear risk of bias	Unclear risk of bias	Stated but not tested	Stated but not tested	Unclear risk of bias	Unclear risk of bias	Unclear risk of bias
49.	NCT01020799	Unclear risk of bias	Unclear risk of bias	Stated but not tested	Stated but not tested	Stated but not tested	Low risk of bias	Low risk of bias
50.	NCT01808612	Unclear risk of bias	Unclear risk of bias	Stated but not tested	Stated but not tested	Stated but not tested	Low risk of bias	Low risk of bias
51.	Nierenberg 2007	Low risk of bias	Low risk of bias	Stated but not tested	Stated but not tested	Unclear risk of bias	Low risk of bias	Unclear risk of bias
52.	NKD20006	Unclear risk of bias	Low risk of bias	Stated but not tested	Stated but not tested	Unclear risk of bias	Low risk of bias	High risk of bias
53.	PAR 279 MDUK	Unclear risk of bias	Unclear risk of bias	Stated but not tested	Stated but not tested	Unclear risk of bias	Low risk of bias	Unclear risk of bias
54.	Perahia 2006	Unclear risk of bias	Unclear risk of bias	Stated but not tested	Stated but not tested	Unclear risk of bias	Low risk of bias	Low risk of bias

55.	**Perahia 2008b**	Unclear risk of bias	Unclear risk of bias	Stated but not tested	Stated but not tested	Unclear risk of bias	Low risk of bias	Unclear risk of bias
56.	**Rapaport 2009**	Unclear risk of bias	Unclear risk of bias	Stated but not tested	Stated but not tested	Unclear risk of bias	Low risk of bias	Unclear risk of bias
57.	**Rudolph 1998**	Low risk of bias	Unclear risk of bias	Stated but not tested	Stated but not tested	Unclear risk of bias	Low risk of bias	Unclear risk of bias
58.	**SCT-MD-35**	Unclear risk of bias	Unclear risk of bias	Stated but not tested	Stated but not tested	Unclear risk of bias	Low risk of bias	Unclear risk of bias
59.	**SCT-MD-49**	Unclear risk of bias	Unclear risk of bias	Stated but not tested	Stated but not tested	Stated but not tested	Low risk of bias	Unclear risk of bias
60.	**SER 101**	Unclear risk of bias	Unclear risk of bias	Stated but not tested	Stated but not tested	Unclear risk of bias	High risk of bias	High risk of bias
61.	**SER 310**	Unclear risk of bias	Unclear risk of bias	Stated but not tested	Stated but not tested	Unclear risk of bias	High risk of bias	Unclear risk of bias
62.	**Sramek 1995**	Unclear risk of bias	Unclear risk of bias	Stated but not tested	Stated but not tested	Unclear risk of bias	Low risk of bias	Low risk of bias
63.	**Study 62a**	Unclear risk of bias	Unclear risk of bias	Stated but not tested	Stated but not tested	Unclear risk of bias	Low risk of bias	High risk of bias
64.	**Study 62b**	Unclear risk of bias	Unclear risk of bias	Stated but not tested	Stated but not tested	Unclear risk of bias	Low risk of bias	High risk of bias
65.	**Study 89306**	Unclear risk of bias	Unclear risk of bias	Stated but not tested	Stated but not tested	Unclear risk of bias	Unclear risk of bias	Unclear risk of bias
66.	**Study F1J-MC-HMAQ-Study Group B**	Unclear risk of bias	Unclear risk of bias	Stated but not tested	Stated but not tested	Unclear risk of bias	Low risk of bias	Unclear risk of bias
67.	**Trivedi 2004**	Unclear risk of bias	Unclear risk of bias	Stated but not tested	Stated but not tested	Unclear risk of bias	Low risk of bias	Unclear risk of bias
68.	**VEN 600A-313**	Unclear risk of bias	Unclear risk of bias	Stated but not tested	Stated but not tested	Unclear risk of bias	Low risk of bias	Unclear risk of bias
69.	**VEN XR 367**	Unclear risk of bias	Unclear risk of bias	Stated but not tested	Stated but not tested	Unclear risk of bias	Low risk of bias	High risk of bias
70.	**Wade 2002**	Unclear risk of bias	Unclear risk of bias	Stated but not tested	Stated but not tested	Unclear risk of bias	Low risk of bias	Low risk of bias
71.	**Wernicke1988**	Unclear risk of bias	Unclear risk of bias	Stated but not tested	Stated but not tested	Stated but not tested	High risk of bias	Unclear risk of bias
72.	**Yevtushenko 2007**	Unclear risk of bias	Unclear risk of bias	Stated but not tested	Stated but not tested	Stated but not tested	Unclear risk of bias	Low risk of bias
73.	**003-008**	Unclear risk of bias	Unclear risk of bias	Stated but not tested	Stated but not tested	Stated but not tested	Low risk of bias	High risk of bias
74.	**003-042**	Unclear risk of bias	Unclear risk of bias	Stated but not tested	Stated but not tested	Stated but not tested	Low risk of bias	High risk of bias
75.	**003-048**	Unclear risk of bias	Unclear risk of bias	Stated but not tested	Stated but not tested	Stated but not tested	Low risk of bias	High risk of bias
76.	**245 (EMD 68 843-010)**	Unclear risk of bias	Unclear risk of bias	Stated but not tested	Stated but not tested	Unclear risk of bias	Unclear risk of bias	Unclear risk of bias
77.	**246 (SB 659746-003)**	Unclear risk of bias	Unclear risk of bias	Stated but not tested	Stated but not tested	Unclear risk of bias	Unclear risk of bias	Unclear risk of bias

4 *Dose-outcome curves*

eFigure 1. Dose-outcome relationships for individual SSRI

	Response	Dropout due to adverse effects	Dropout for any reason
**Citalopram (17 arms)**	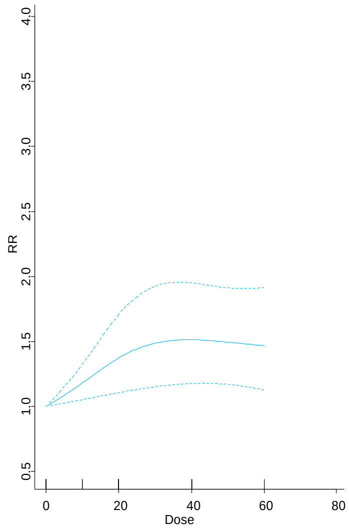	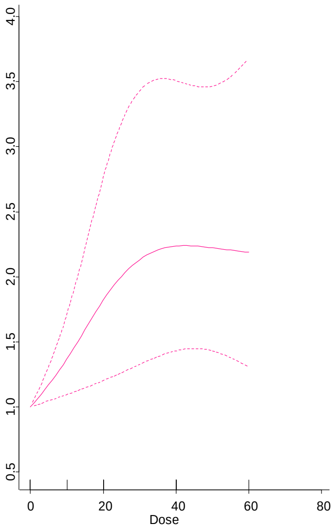	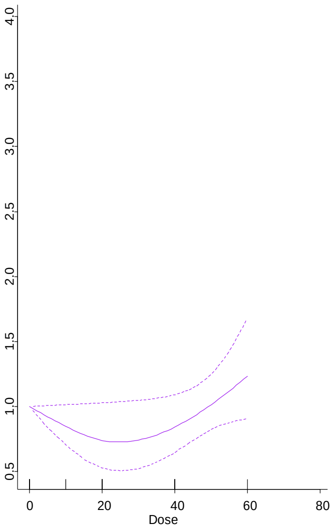
**Escitalopram (16 arms)**	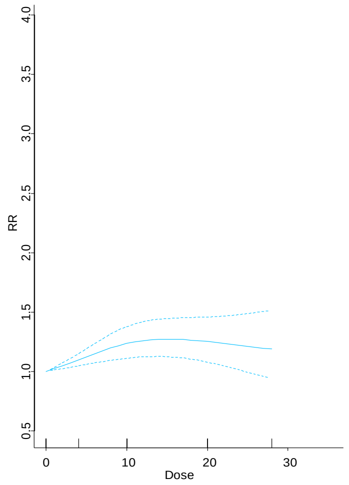	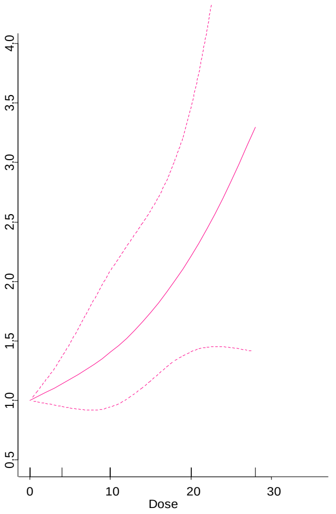	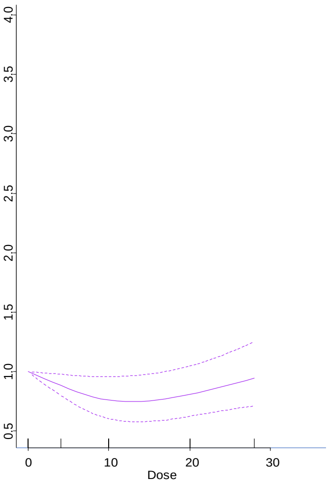

Fluoxetine

(27 arms)

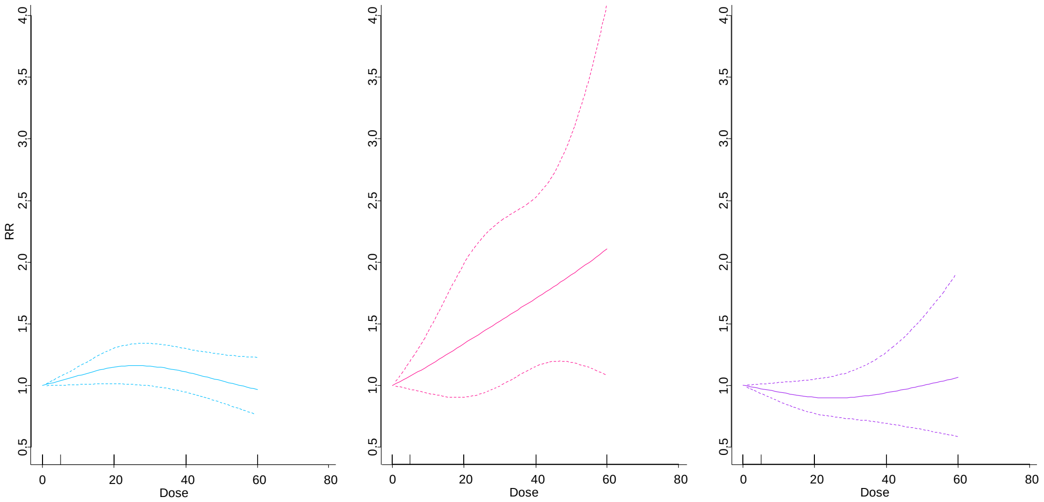

Paroxetine

(28 arms)

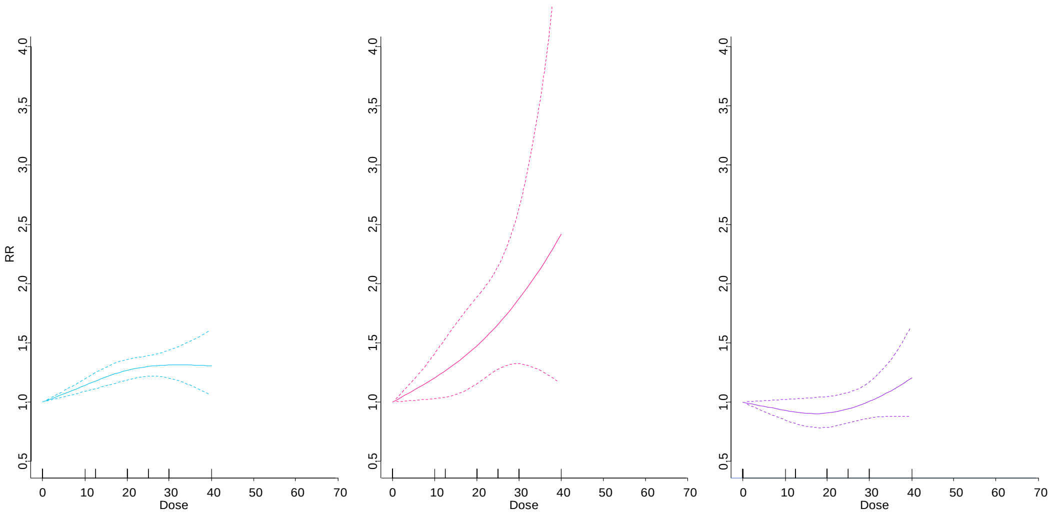

Sertraline

(11 arms)

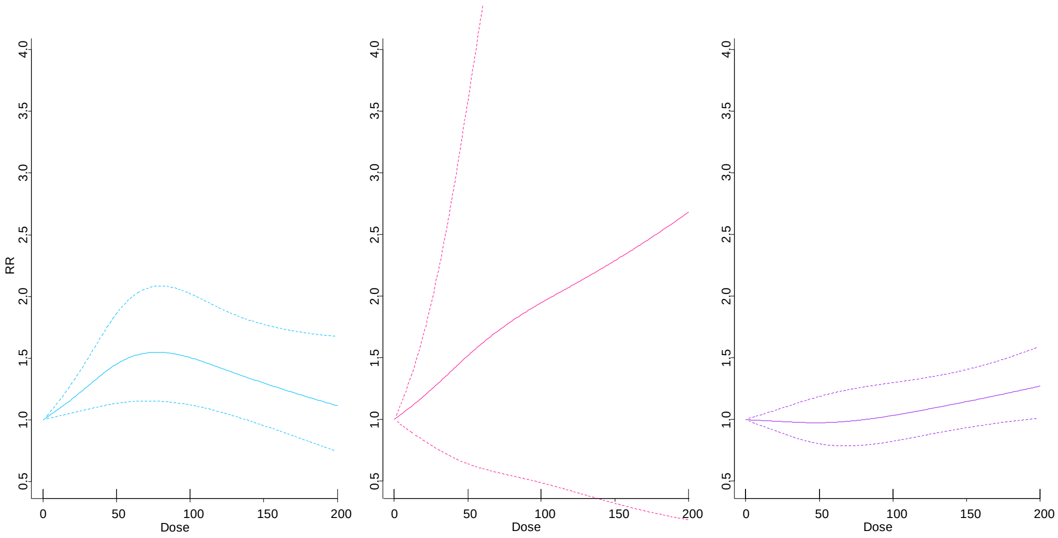

Each tick on the horizontal axis represents the dosage examined in a study arm.

The dotted lines represent 95% confidence intervals.

eFigure 2. Spline curves using various knots and their goodness-of-fit

Knots	Response	Dropouts due to adverse effects	Dropouts for any reason
**(20,30,40)**	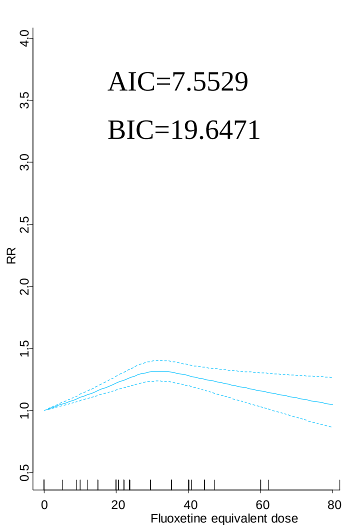	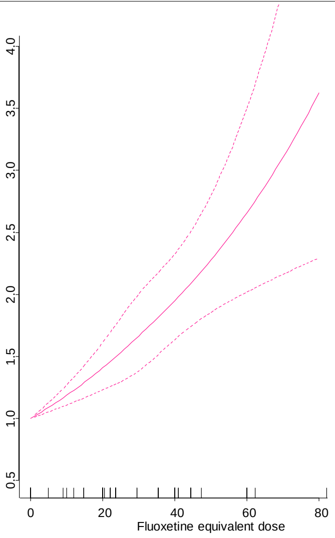	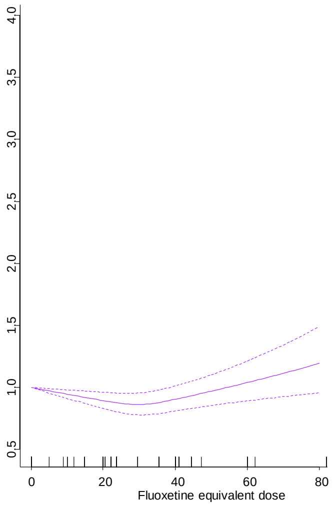
**(10,30,50)**	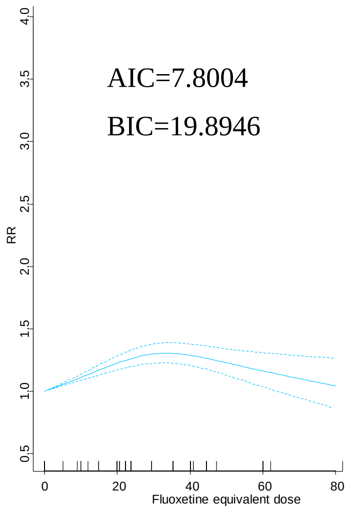	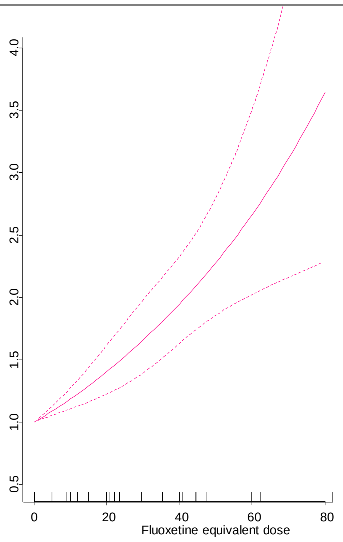	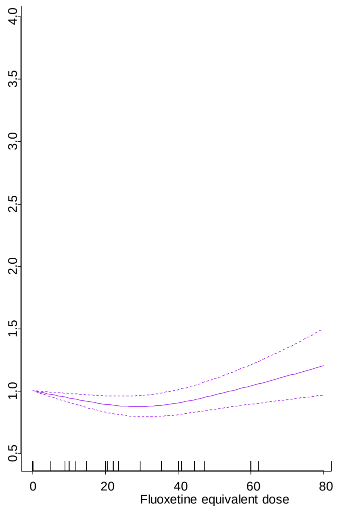

(10,20,40)

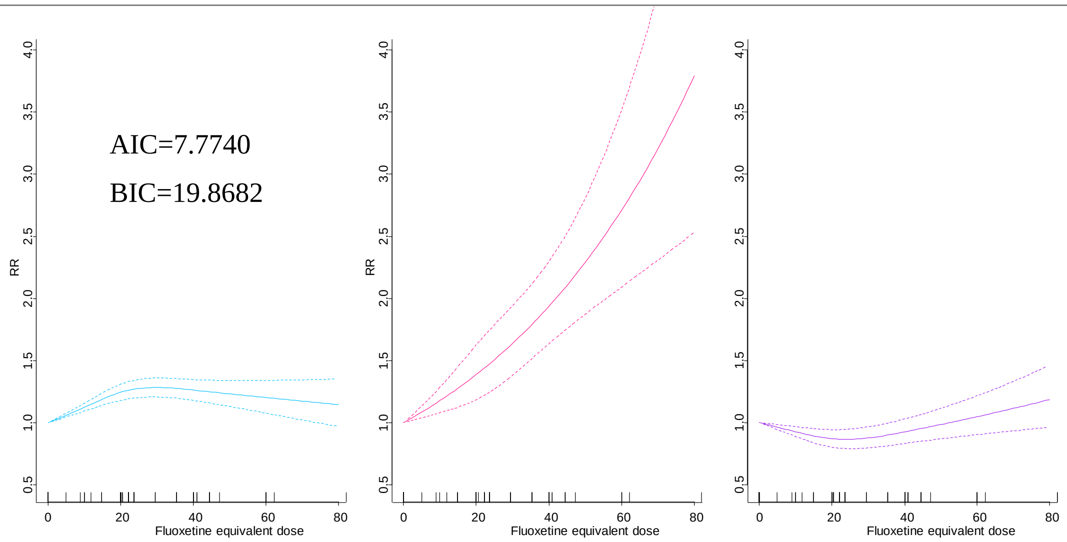

(20,40,60)

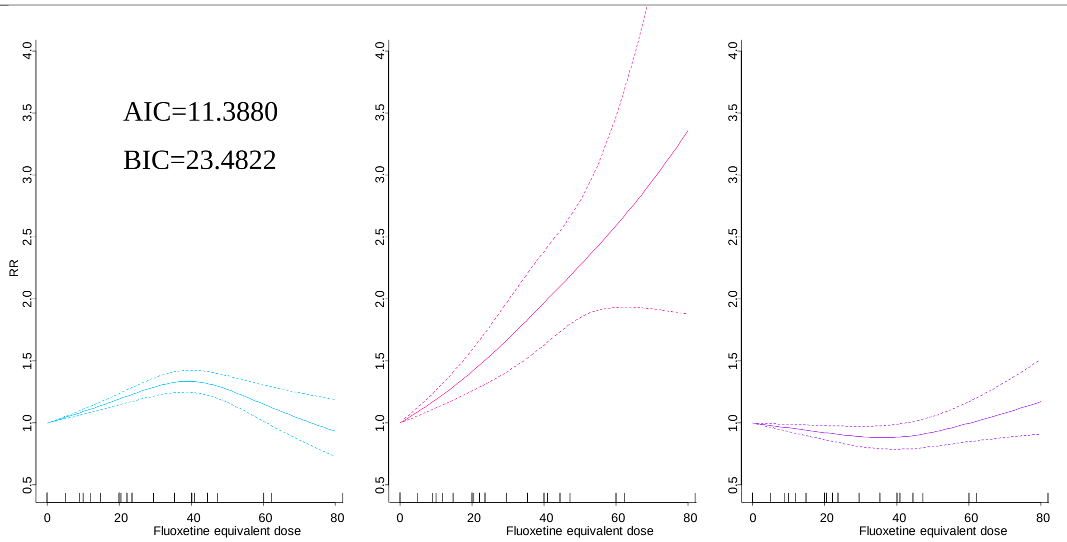

(10,20,50)

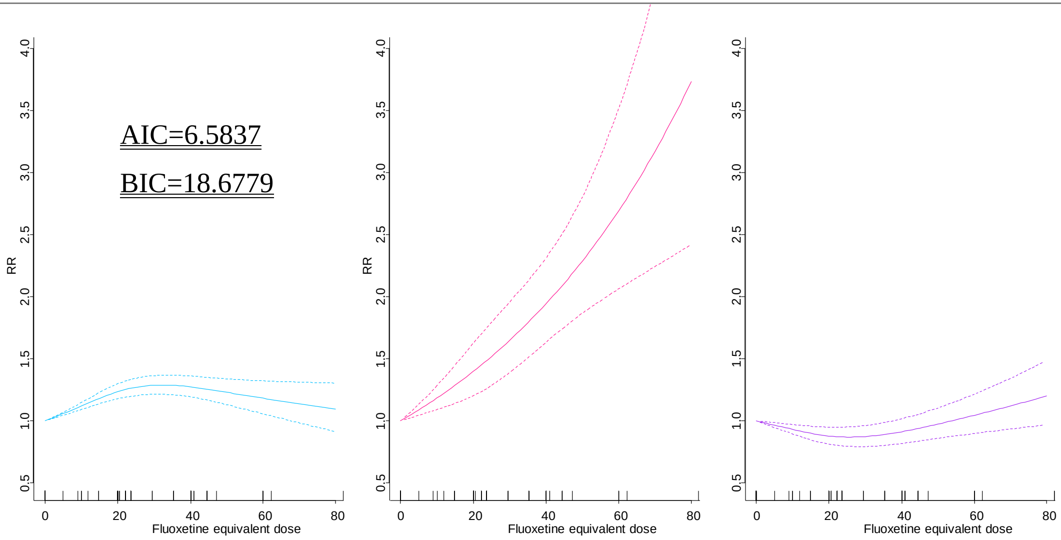

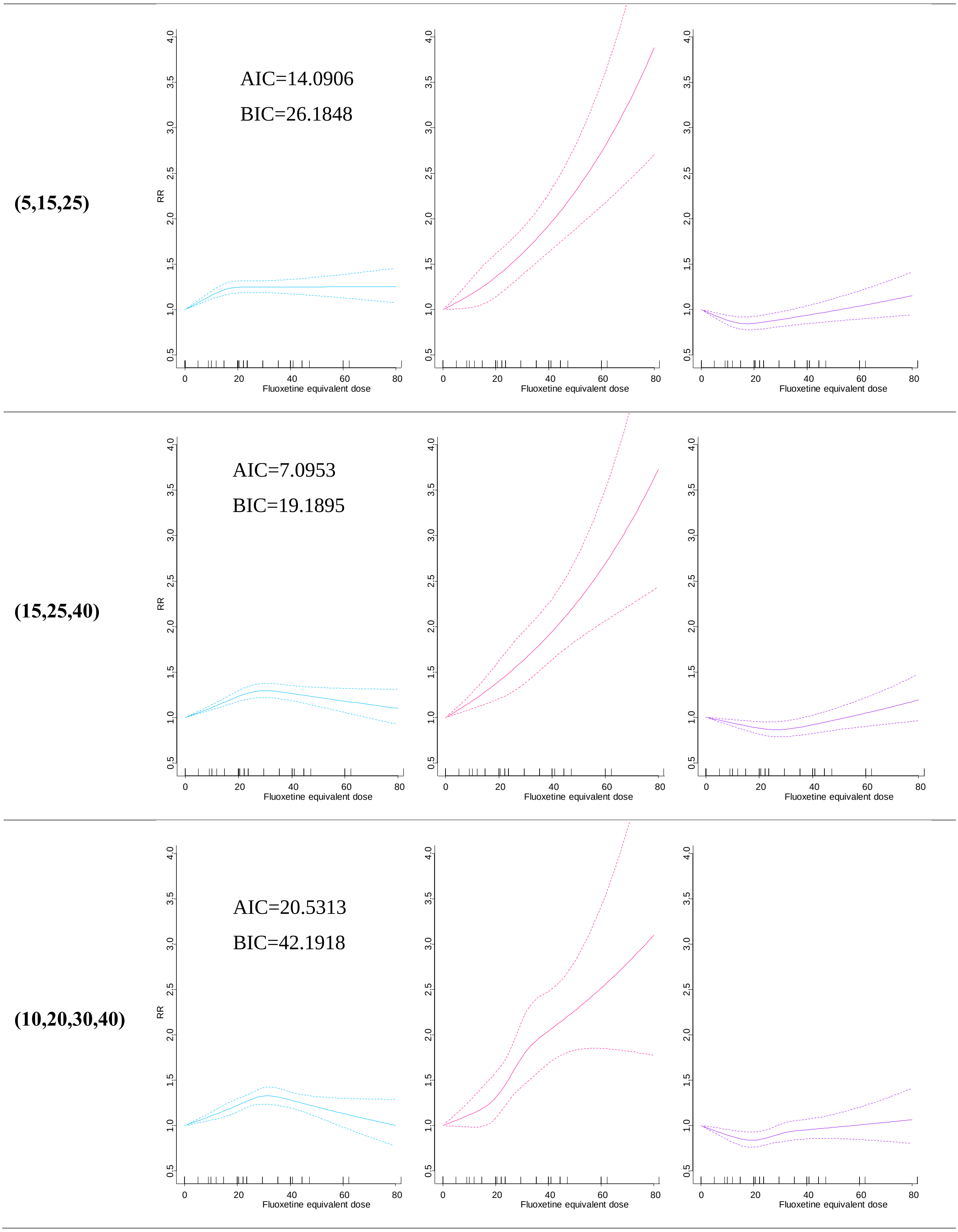

Each tick on the horizontal axis represents the dosage examined in a study arm.

The dotted lines represent 95% confidence intervals.

AIC: Akaike Information Criterion, BIC: Baeysian Information Criterion

The double underline shows the model with the best fit.

eFigure 3. Dose-outcome relationships using different dose conversion algorithms

Dose conversion algorithm	Response	Dropout due to adverse effects	Dropout for any reason
**Jakubovski**	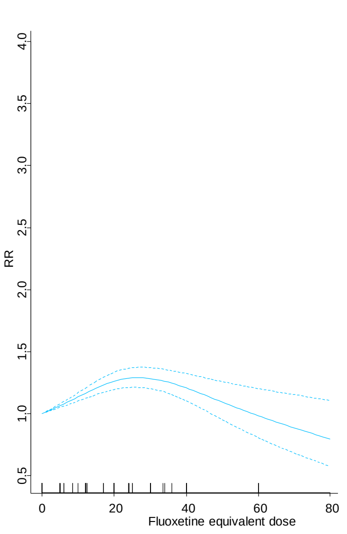	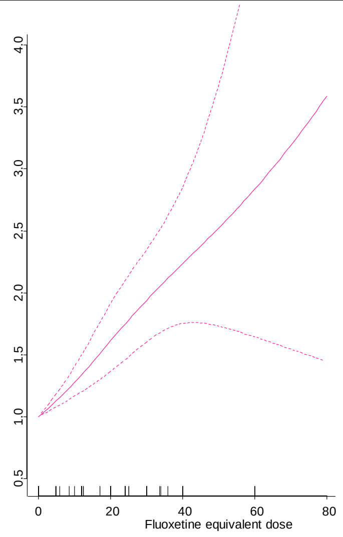	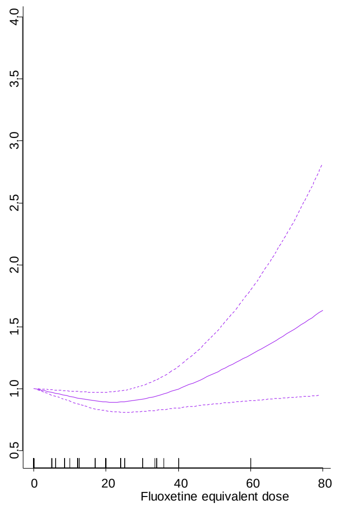
**MEPS**	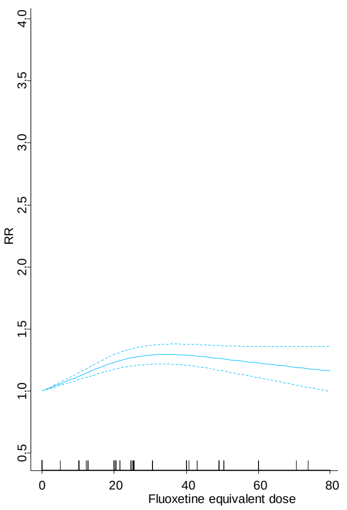	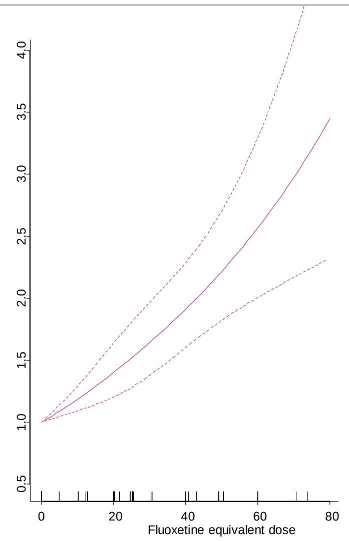	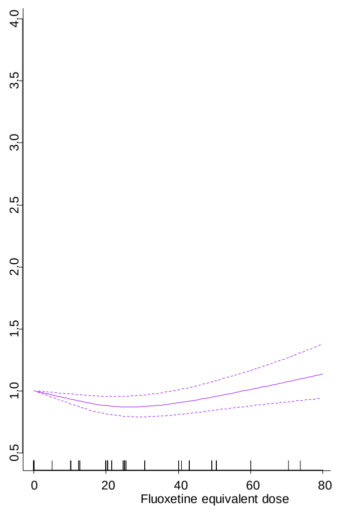

Each tick on the horizontal axis represents the dosage examined in a study arm.

The dotted lines represent 95% confidence intervals.

MEPS: Medical Expenditure Panel Survey

eFigure 4. Dose-outcome relationships among studies at low risk of bias only

Response	Dropout due to adverse effects	Dropout for any reason
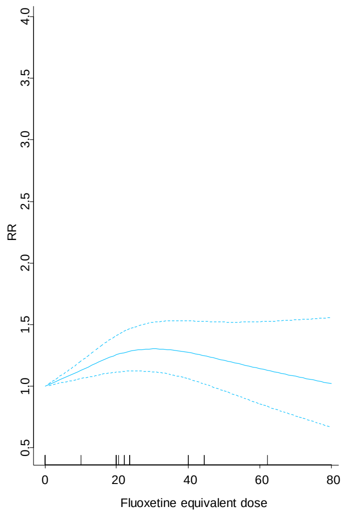	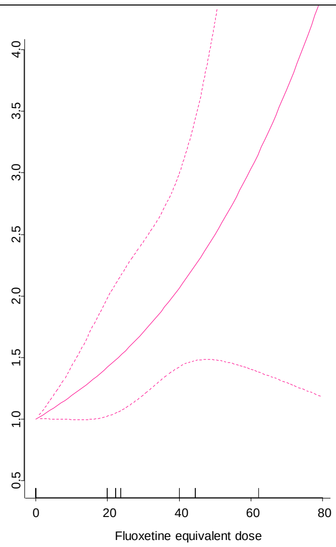	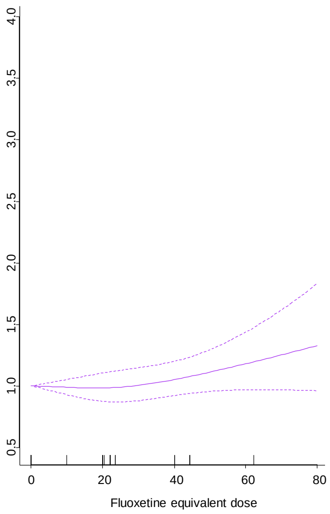

Each tick on the horizontal axis represents the dosage examined in a study arm.

The dotted lines represent 95% confidence intervals.

eFigure 5. Dose-remission relationship for SSRIs, venlafaxine and mirtazapine

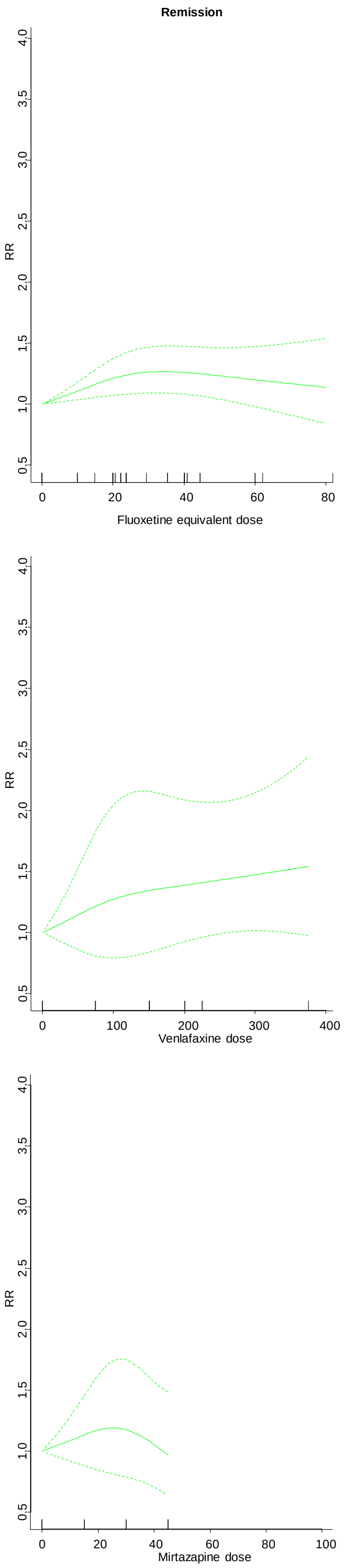

Each tick on the horizontal axis represents the dosage examined in a study arm.

The dotted lines represent 95% confidence intervals.
